# Activation of E2F-dependent transcription by the mouse cytomegalovirus M117 protein affects the viral host range

**DOI:** 10.1371/journal.ppat.1007481

**Published:** 2018-12-10

**Authors:** Eléonore Ostermann, Stefan Loroch, Zhikang Qian, Albert Sickmann, Lüder Wiebusch, Wolfram Brune

**Affiliations:** 1 Heinrich Pette Institute, Leibniz Institute for Experimental Virology, Hamburg, Germany; 2 Leibniz-Institut für Analytische Wissenschaften – ISAS – e.V., Dortmund, Germany; 3 Unit of Herpesvirus and Molecular Virology, Key Laboratory of Molecular Virology & Immunology, Institut Pasteur of Shanghai, Chinese Academy of Sciences, Shanghai, China; 4 Labor für Pädiatrische Molekularbiologie, Charité Universitätsmedizin Berlin, Berlin, Germany; University of Wisconsin-Madison, UNITED STATES

## Abstract

Cytomegaloviruses (CMVs) have a highly restricted host range as they replicate only in cells of their own or closely related species. To date, the molecular mechanisms underlying the CMV host restriction remain poorly understood. However, it has been shown that mouse cytomegalovirus (MCMV) can be adapted to human cells and that adaptation goes along with adaptive mutations in several viral genes. In this study, we identify MCMV M117 as a novel host range determinant. Mutations in this gene enable the virus to cross the species barrier and replicate in human RPE-1 cells. We show that the M117 protein is expressed with early kinetics, localizes to viral replication compartments, and contributes to the inhibition of cellular DNA synthesis. Mechanistically, M117 interacts with members of the E2F transcription factor family and induces E2F target gene expression in murine and human cells. While the N-terminal part of M117 mediates E2F interaction, the C-terminal part mediates self-interaction. Both parts are required for the activation of E2F-dependent transcription. We further show that M117 is dispensable for viral replication in cultured mouse fibroblasts and endothelial cells, but is required for colonization of mouse salivary glands in vivo. Conversely, inactivation of M117 or pharmacological inhibition of E2F facilitates MCMV replication in human RPE-1 cells, whereas replacement of M117 by adenovirus E4orf6/7, a known E2F activator, prevents it. These results indicate that E2F activation is detrimental for MCMV replication in human cells. In summary, this study identifies MCMV M117 as a novel E2F activator that functions as a host range determinant by precluding MCMV replication in human cells.

## Author summary

Human CMV is an opportunistic pathogen causing morbidity and mortality in immunocompromised individuals. It is a highly species-specific virus that replicates only in cells from humans or chimpanzees, but not in cells from mice or other laboratory animals. Mouse cytomegalovirus (MCMV), the most commonly used model to study CMV pathogenesis in vivo, is also species-specific and does not replicate in human cells. However, the causes of the CMV host species specificity have remained largely unknown. Here we show that the viral M117 protein is a major factor contributing to the MCMV host species specificity. When M117 is inactivated, MCMV acquires the ability to replicate in human cells. We further demonstrate that M117 interacts with transcription factors of the E2F family and activates E2F-dependent gene expression. While this function is needed for MCMV dissemination in mice, it is detrimental for MCMV replication in human cells. The results of this study indicate that the host range of a virus, i.e. its ability to replicate in cells from different hosts, can depend on an appropriate regulation of transcription factors.

## Introduction

Viruses are obligate intracellular parasites. As such, they rely on suitable host cells for their replication. While some viruses can infect and replicate in cells from various tissues and different host species, others are highly adapted to their natural host and have a narrow host range [1,2].

Cytomegaloviruses (CMVs), representatives of the β-herpesvirus subfamily, are highly species-specific as they can replicate only in cells of their own or closely related host species [3]. Human CMV (HCMV), an opportunistic pathogen causing morbidity and mortality in immunocompromised individuals, replicates in cells from humans or chimpanzees, but not in cells from mice or other small animals. Consequently, HCMV pathogenesis cannot be studied in small animal models. Instead, related viruses such as the mouse and rat cytomegaloviruses (MCMV and RCMV) are used as models to study CMV pathogenesis in their natural hosts. Conversely, these viruses do not replicate in human cells. Early attempts to understand the CMV host species specificity have revealed that the CMVs can enter non-permissive host cells and even express a subset of viral genes, mainly of the immediate early (IE) class, but viral DNA replication and late gene expression are inefficient or absent [4,5]. Hence the CMV host cell restriction is thought to be caused by a post-penetration block to viral gene expression and replication [6]. However, the molecular mechanisms underlying the CMV host species specificity have remained largely unknown.

A few studies have shown that MCMV is somewhat less restricted in its host range than HCMV [5,7]. An important restriction to MCMV replication in human cells is infection-induced apoptosis and the inability of MCMV to inhibit it in human cells. Enforced inhibition of apoptosis was sufficient to allow MCMV replication in human cells [8]. However, MCMV replication under these conditions was slow and inefficient, suggesting that additional checkpoints and restrictions exist. Other studies suggested that the virus’ ability to counteract suppression by PML nuclear bodies might play an important role in the viral host range [9,10].

An attractive and promising strategy to identify novel host range determinants is to characterize mutations in viruses adapted to cells of a foreign host [1,11]. Although the first report of a spontaneous MCMV adaptation to human cells was published almost 50 years ago [12], identification and verification of adaptive mutations within the 230 kbp MCMV genome have become possible only recently with the availability of cost-efficient high-throughput sequencing [13,14]. The first MCMV host range factor identified with this strategy was the viral M112-113 gene [13].

One important difference between HCMV and MCMV is their dependence on the cell cycle stage. HCMV-infected cells need to be in the G1 phase in order to express viral IE genes [15,16], whereas the expression of MCMV genes is independent of the cell cycle phase [17]. To facilitate viral DNA replication, these viruses arrest the cell cycle at the G1/S transition [18–23], where the components necessary for viral replication are available, while blocking cellular DNA synthesis [24–26].

The E2F transcription factors were first described in 1986 as DNA binding proteins activating the adenoviral E2a promoter [27]. Since then, eight E2F proteins (E2F1-8) have been discovered in mammalian cells and classified either as transcriptional activators (E2F1-3) or repressors (E2F4-8) (reviewed in [28,29]). However, this classification is probably oversimplified as some activators can also repress gene expression [30] while the repressors, such as E2F4, can activate the expression of certain target genes [31]. E2F1-6 transcription factors form heterodimers with dimerization partner proteins DP1 or DP2 that stabilize the DNA binding of the complex and target the transcription factors to specific promoters [32]. The transcription factor activity of the E2F family is negatively regulated by their association with cell cycle regulators of the retinoblastoma protein family (pRb, p107, and p130), also known as pocket proteins. Phosphorylation of the pocket proteins releases the E2F-DP complex, which can then activate the expression of the E2F-dependent genes [33]. Several studies have shown that E2F activation promotes cell cycle progression into S phase, as many E2F target genes are involved in the process of DNA replication [34].

Some viruses, such as adeno-, papilloma-, and polyomaviruses promote E2F-dependent transcription and cell cycle progression towards S phase through viral proteins (E1A, E7, and large T, respectively) [35] that disrupt the interaction between the pocket proteins and E2F. Similarly, the HCMV pp71 and pUL97 proteins regulate the Rb-E2F interaction by several distinct mechanisms [36–38]. Only a few viral proteins interact directly with E2F proteins and regulate their activity. The most studied is E4orf6/7 from adenovirus (AdV) type 5, which was shown to interact with all canonical E2F members and activate E2F-dependent transcription [39–41]. Other viral proteins, such as HCMV IE1, HPV16 E7 and the HIV-1 Tat protein activate E2F-dependent transcription via an interaction with only one E2F member [42–44]. It has also been shown that the CMVs transactivate the expression of the cellular genes involved in DNA replication in an E2F-dependent manner [45–47].

Here we show that the MCMV M117 protein interacts with all canonical E2F family members and activates E2F-dependent gene expression in MCMV-infected cells. M117 inactivation did not impair MCMV replication in murine cells, but massively reduced viral dissemination to the salivary glands of infected mice. By specific mutagenesis of M117 we showed that the interaction with E2F3 is of particular importance for transcriptional activation of target genes and for viral dissemination of MCMV in vivo. Intriguingly, M117 inactivation or pharmacological inhibition of E2F-dependent transcription facilitated MCMV replication in human cells while expression of the AdV E4orf6/7 protein, a known E2F activator, inhibited MCMV replication, suggesting that some E2F target proteins in human cells restrict MCMV replication.

## Results

### M117 is a host range determinant

**Fig 1:**
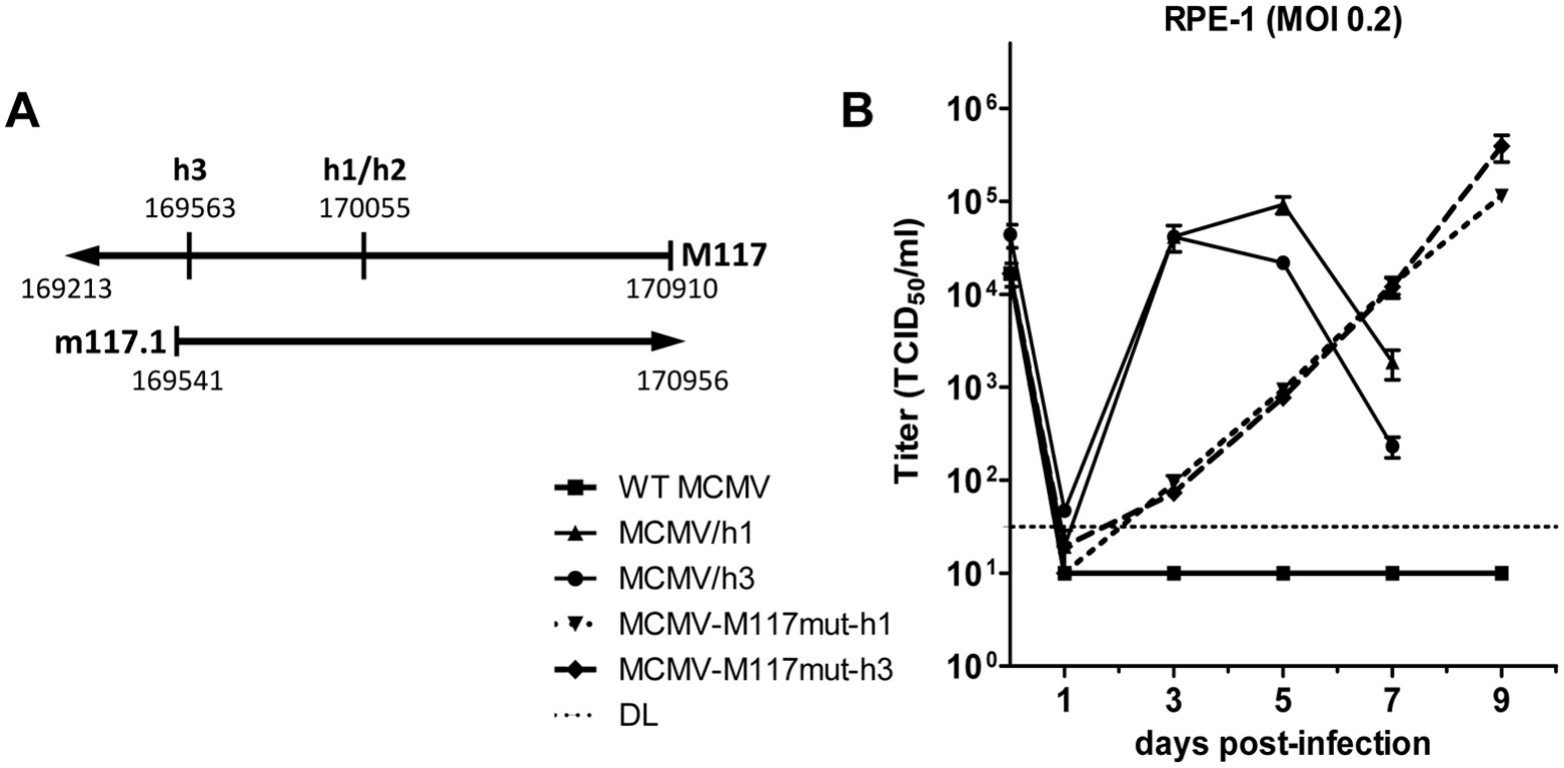
Mutations in M117 facilitate MCMV replication in human cells. (A) Schematic representation of the M117 region with the nucleotide position of each ORF in the MCMV Smith sequence (NC_004065). The positions of the h1/h2 and h3 mutations are indicated. (B) Multistep replication kinetics in human RPE-1 cells. Cells were infected with WT MCMV, the human cell-adapted MCMV/h1 and MCMV/h3 as well as the single M117 mutants MCMV-M117mut-h1 and MCMV-M117mut-h3 by using an MOI of 0.2 TCID_50_/cell. The experiment was done in triplicate. Viral titers were determined in the supernatant and are shown as mean ±SEM. DL, detection limit.

In previous work we have reported the isolation of three human cell-adapted MCMVs (MCMV/h1, MCMV/h2, and MCMV/h3) and described the mutations associated with adaptation to human RPE-1 epithelial cells [14]. All three adapted MCMVs carried mutations within ORF M117, which were predicted to cause expression of truncated M117 proteins (Fig 1A): in MCMV/h1 and /h2 the same point mutation (C to A) leads to the introduction of a premature stop codon, and in MCMV/h3 a single nucleotide deletion (ΔT) leads to a frameshift. We hypothesized that these mutations contributed to human cell adaptation and that M117 is a host range determinant. To test this hypothesis, the two different mutations were introduced individually into MCMV-GFP, an MCMV strain expressing the enhanced green fluorescent protein. The resulting mutant viruses were named MCMV-M117mut-h1 and MCMV-M117mut-h3, respectively. Multistep replication kinetics in human RPE-1 epithelial cells were analyzed with both recombinant viruses. While the parental WT MCMV did not replicate in human RPE-1 cells, both MCMV-M117mut-h1 and MCMV-M117mut-h3 viruses replicated to high titers in human cells (Fig 1B). However, compared to the spontaneously adapted viruses MCMV/h1 and MCMV/h3, the constructed M117 mutants reached their peak titers several days later, indicating that additional mutations present in MCMV/h1, MCMV/h2, and MCMV/h3 [14] contributed to human cell adaptation.

### M117 is an early protein localizing to intranuclear replication compartments

According to the MCMV genome annotation by Rawlinson [48] the M117 ORF overlaps with the m117.1 ORF encoded on the opposite strand (Fig 1A). Which of the two ORFs is transcribed and translated into a protein has not been established yet. However, low levels of M117 transcripts, but not of m117.1 transcripts, were detected by RNA sequencing and ribosome profiling studies [49,50]. Another study reported detection of low levels of m117.1 transcripts [51]. Moreover, m117.1-derived peptide fragments have been detected in purified MCMV virions by mass spectrometry [52].

**Fig 2:**
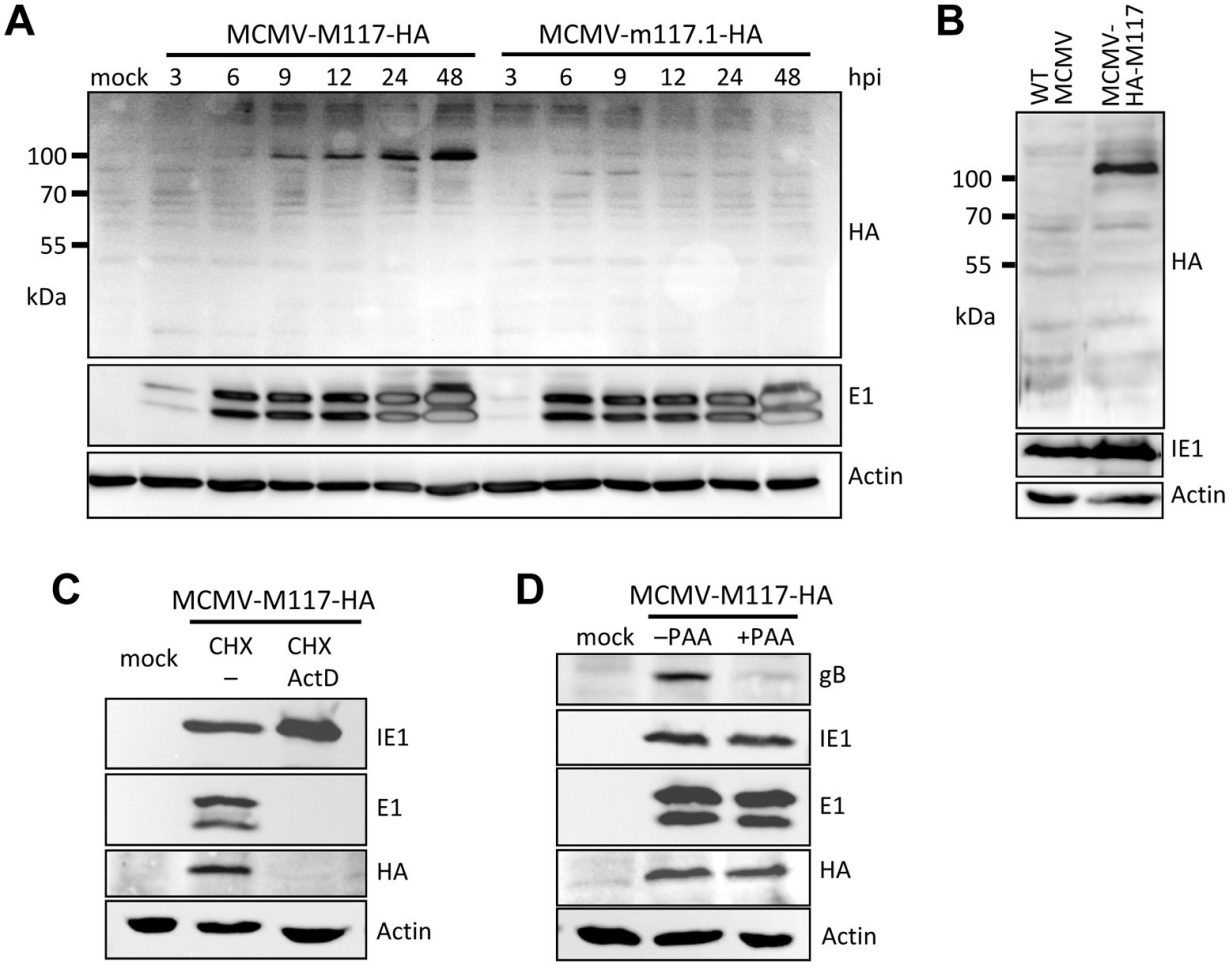
M117 is an early protein. (A) Murine 10.1 fibroblasts were infected with the 3ˈ M117 or m117.1 HA-tagged viruses at an MOI of 1 TCID_50_/cell. Cell lysates were harvested at the indicated times post infection and subjected to SDS-PAGE. 20 μg of protein were loaded. MCMV E1 and cellular β-actin served as infection and loading controls, respectively. (B) Murine 10.1 fibroblasts were infected with the WT MCMV or the 5' M117 HA-tagged viruses at an MOI of 1 TCID_50_/cell and cell lysates were harvested 24 hpi and subjected to SDS-PAGE. (C) Cells infected with MCMV-M117-HA were treated for 4 hours with 50 μg/mL cycloheximide (CHX) followed by 5 μg/mL Actinomycin D (ActD) or no (–) treatment for 7.5 hours prior to western blot analysis. (D) Cells infected with MCMV-M117-HA were treated for 24 hours with 250 ng/mL phosphonoacetic acid (PAA) prior to Western blot analysis.

To determine whether MCMV expresses M117 or m117.1 proteins during infection, we constructed recombinant MCMV viruses carrying an HA tag at the 3ˈ end of ORFs M117 or m117.1, respectively. The MCMV-M117-HA and MCMV-m117.1-HA viruses were used to infect murine fibroblasts, and expression of HA-tagged proteins was assessed by Western blot. In cells infected by MCMV-M117-HA, a protein with a molecular weight of approximately 100 kDa could be detected as early as 6 hours post infection (hpi) (Fig 2A). The M117 protein was apparently expressed at a low level as its detection by Western blot required a long exposure. This is consistent with the low transcript levels detected in previous studies [49,50]. In contrast, no specific HA-tagged protein could be detected in cells infected with MCMV-m117.1-HA (Fig 2A). The apparent molecular weight of M117-HA (100 kDa) differed from its predicted molecular weight (62 kDa), suggesting posttranslational modifications of the M117 protein or expression of a protein product from a larger, possibly spliced, transcript. To exclude the latter, another recombinant MCMV carrying an HA tag sequence at the 5' end of M117 (MCMV-HA-M117) was constructed. As shown in Fig 2B, fibroblasts infected by MCMV-HA-M117 expressed an HA-tagged protein of approx. 100 kDa, thus suggesting that the mass increase was due to posttranslational modifications rather than expression of a larger spliced transcript. It is also noteworthy that two closely related virus species, the Maastricht and English isolates of rat CMVs (murid herpesviruses 2 and 8) contain ORFs homologous to M117, but not to m117.1.

The M117 protein expression time course (Fig 2A) suggested that M117 belongs to the early (β) kinetic class. To verify this, we used a cycloheximide (CHX) release assay that allows selective expression of viral immediate early (α) genes. Cells were infected in the presence of CHX to allow α gene transcription but not translation. After 4 hours, the CHX-containing medium was replaced by either normal medium or actinomycin D (ActD)-containing medium. The removal of CHX allowed the synthesis of proteins from immediate early transcripts while β gene transcription was blocked by ActD. As shown in Fig 2C, the MCMV immediate-early 1 (IE1) protein was expressed with immediate early kinetics, but the viral early 1 (E1, M112-113) proteins and M117 were not. We also tested the expression of M117 in the presence of phosphonoacetic acid (PAA), an inhibitor of viral DNA replication and late (γ) protein expression. PAA inhibited the expression of the MCMV late protein gB, but not the expression of IE1, E1, and M117 (Fig 2D). Taken together these results demonstrated that M117 is expressed with early (β) kinetics.

**Fig 3:**
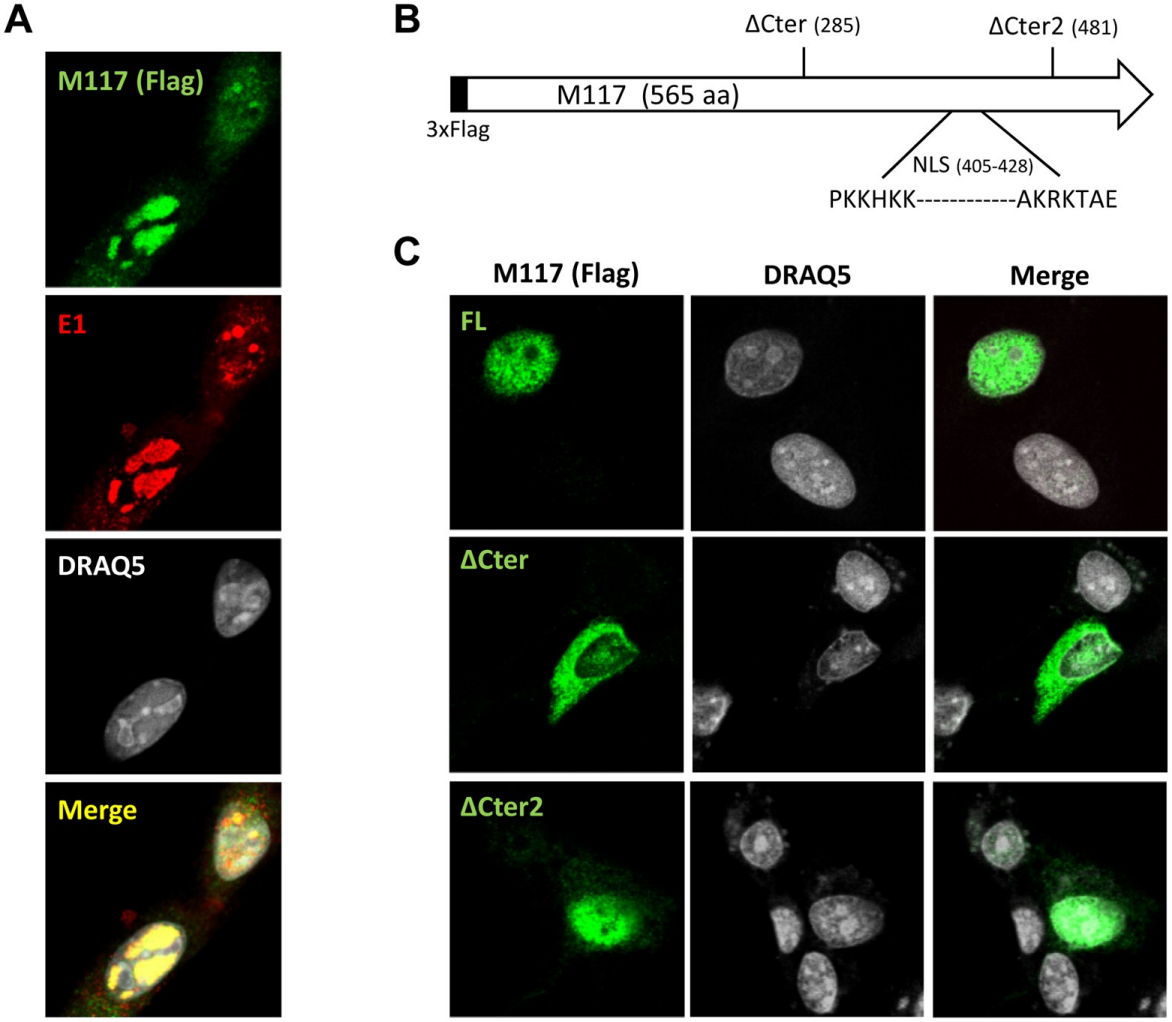
M117 localizes to viral replication compartments in the nucleus. (A) Immunofluorescence of 10.1 fibroblasts infected with MCMV-3xFlag-M117 (MOI of 1 TCID_50_/cell). Cells were fixed 24 hpi and stained with antibodies raised against Flag and the MCMV E1 protein, a marker for viral replication compartments. Nuclei were stained with DRAQ5. (B) Schematic representation of the M117 protein. The positions of the predicted bipartite NLS, the stop codons of the ΔCter and ΔCter2 mutants are shown. (C) Immunofluorescence of 10.1 fibroblasts 24 hours post transfection with plasmids encoding triple Flag-tagged full-length (FL) M117, or truncated M117-ΔCter and M117-ΔCter2 proteins.

Next, we wanted to determine the intracellular localization of the M117 protein during MCMV infection. Initial experiments performed with MCMV-HA-M117 did not yield satisfactory results as the immunofluorescence (IF) signals were very weak due to the low expression level of M117. To increase signal intensity, a mutant virus carrying a triple Flag tag at the N-terminus of M117 (MCMV-M117-FL) was created. During infection, the 3xFlag-tagged M117 protein could be detected in dot-like structures in the nuclei of infected cells. These dots colocalized with the viral E1 proteins (Fig 3A), which are markers for viral replication compartments [53,54]. In silico analysis of the M117 amino acid sequence with three different online tools (seqNLS, NUCDISC/PSORTII and ELM) predicted the presence of a bipartite nuclear localization signal (NLS) starting at position 405 (Fig 3B). To test the role of the NLS in M117 nuclear localization, 3xFlag-M117 and the two C-terminal truncation mutants (ΔCter and ΔCter2) were cloned in a pcDNA3 expression plasmid. The ΔCter and ΔCter2 truncated proteins correspond to the truncated M117 proteins encoded by MCMV-M117mut-h1 and MCMV-M117mut-h3, respectively. ΔCter2 retains the predicted NLS, whereas ΔCter does not (Fig 3B). As shown in Fig 3C, both full-length M117 and the ΔCter2 mutant were detected in the cell nucleus whereas ΔCter was detected predominantly in the cytoplasm. We also observed a difference in the nuclear distribution of M117 during infection and transfection, the protein being dispersed in the nucleus upon transfection (Fig 3C) but localizing in specific intranuclear compartments during infection (Fig 3A).

Taken together these findings suggest that M117 uses the predicted NLS for the localization in the cell nucleus but requires additional viral proteins or viral DNA, present during infection and absent in transfected cells, for its recruitment to viral replication compartments.

### M117 shares structural and functional similarities with HCMV UL117

**Fig 4:**
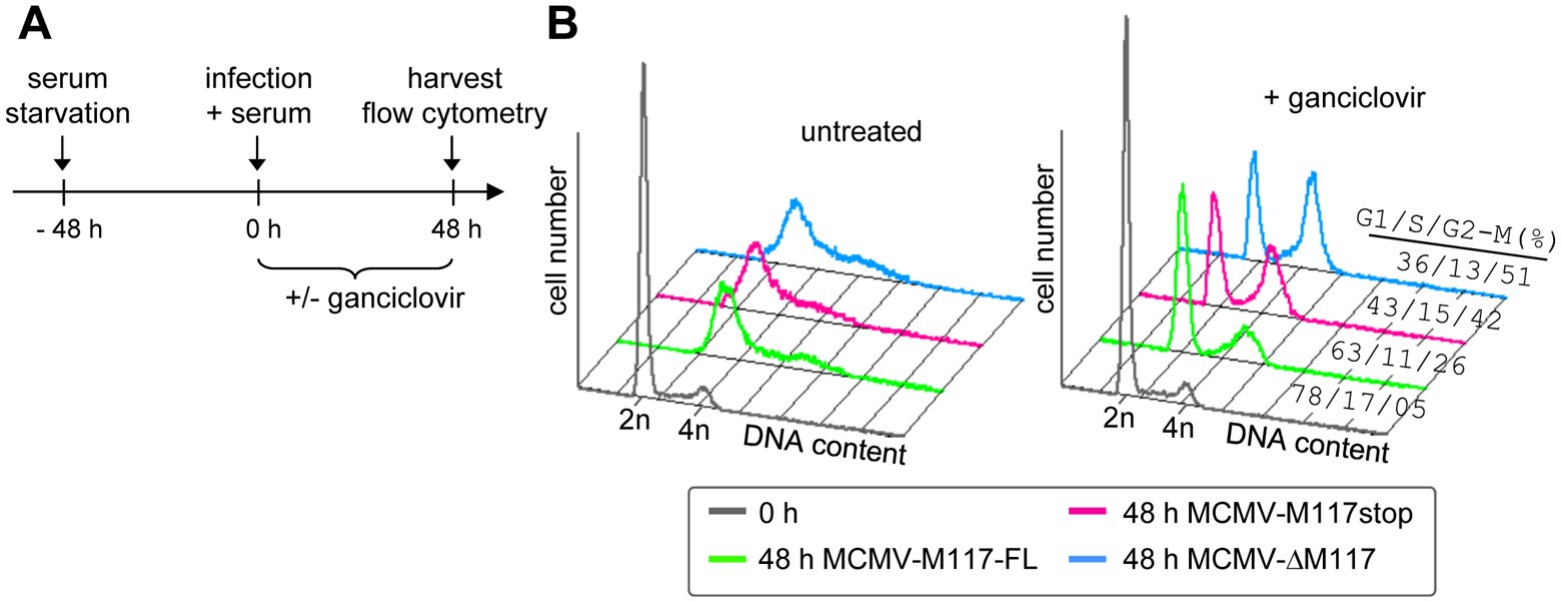
M117 inhibits host DNA synthesis. (A) Experimental setup: NIH-3T3 cells were synchronized in G0/G1 by serum starvation for 48 hours. The infection was performed in the presence of serum at an MOI of 3 in the presence or absence of ganciclovir (GCV, 50 μM). Cells were harvested 48 hpi and stained with propidium iodide and anti-IE1 antibodies prior to flow cytometry. (B) Histogram peaks show the DNA content in IE1 positive gated cells. The cells with a 2n DNA content are in G1 and the 4n content represent the cells in G2/M. Percentages of cells in each phase of the cell cycle are indicated numerically.

The M117 ORF is encoded on the negative strand of the MCMV genome between M116 and M118 [48]. Thus, it is a positional homolog of HCMV UL117, which is also encoded on the negative strand between UL116 and UL118. Although the overall sequence similarity between M117 and pUL117 is low (24%), a sequence alignment of M117 homologs from rodent and primate CMVs revealed stretches of high similarity within the N- and C-terminal parts of the proteins (S1 Fig). Moreover, M117 belongs to the same kinetic class and shows the same nuclear distribution as previously described for the UL117 protein (pUL117) [55]. As pUL117 contributes to cell cycle regulation by suppressing host DNA synthesis [26], we tested whether M117 is also involved in cell cycle regulation. To do this, NIH-3T3 cells were synchronized by 48 hours serum starvation and infected with MCMV expressing M117 (MCMV-M117-FL) or mutants lacking M117 (MCMV-ΔM117 and MCMV-M117stop) for additional 48 hours (Fig 4A). Whereas MCMV-ΔM117 lacks the complete M117 ORF, the MCMV-M117stop virus carries a point mutation that introduces a stop codon at position 21 without changing the amino acid sequence encoded by the m117.1 ORF on the opposite strand. To better recognize a possible effect of M117 on host cell DNA synthesis, viral DNA replication was blocked with Ganciclovir (GCV). Cells were fixed, stained for the viral protein IE1 to identify infected cells and with propidium iodide to analyze DNA content by flow cytometry. As shown in Fig 4B, cells infected with the WT MCMV expressing full length M117 predominantly showed a 2n DNA content consistent with a G1/S cell cycle arrest [17]. Conversely, a larger percentage of cells infected with the viruses lacking M117 had a 4n DNA content (Fig 4B), indicating cell cycle progression through S phase and an arrest at G2/M. These data suggest that M117 has a role in the regulation of host DNA synthesis and cell cycle progression through S phase.

### M117 interacts with E2F transcription factors

In order to understand the mechanism of action of M117, we tried to identify M117-interacting proteins by affinity purification-mass spectrometry (AP-MS). Stable isotope labelling by amino acids in cell culture (SILAC) was used to compare the anti-HA immunoprecipitation products of cells infected with WT MCMV or MCMV-HA-M117. The experiment was done in duplicate including a label switch. Proteins identified by at least 2 unique peptides and a log_2_ ratio ≥ 3 (HA-M117 vs WT) were considered as potential interaction partners. Besides M117 itself, only 4 proteins met these criteria: the viral DNA polymerase processivity factor M44 and three members of the E2F transcription factor family: E2F3, E2F4, and DP1 (Table 1).

**Table 1:** M117 interaction partners identified by AP-MS.

Gene Name	Uniprot Accession	Unique peptide count (Mascot against Uniprot mouse incl. MCMV, FDR ≤ 1% on PSM level)				Enrichment M117-HA vs WT	
		M117-HA	WT	M117-HA	WT	Repl. 1	Repl.2
		(heavy, repl.1)	(light, repl.1)	(light, repl.2)	(heavy, repl.2)	log_2_ (heavy/light)	log_2_ (light/heavy)
M117	A8E1M8	13	2	15	0	5.3	12.8
Tfdp1	Q08639	4	0	3	0	12.7	7.7
E2f3	O35261	4	2	2	0	5.1	8.8
E2f4	Q8R0K9	2	0	2	0	5.8	9.3
M44	A2Q6M6	2	1	7	0	3.4	4.7

**Fig 5:**
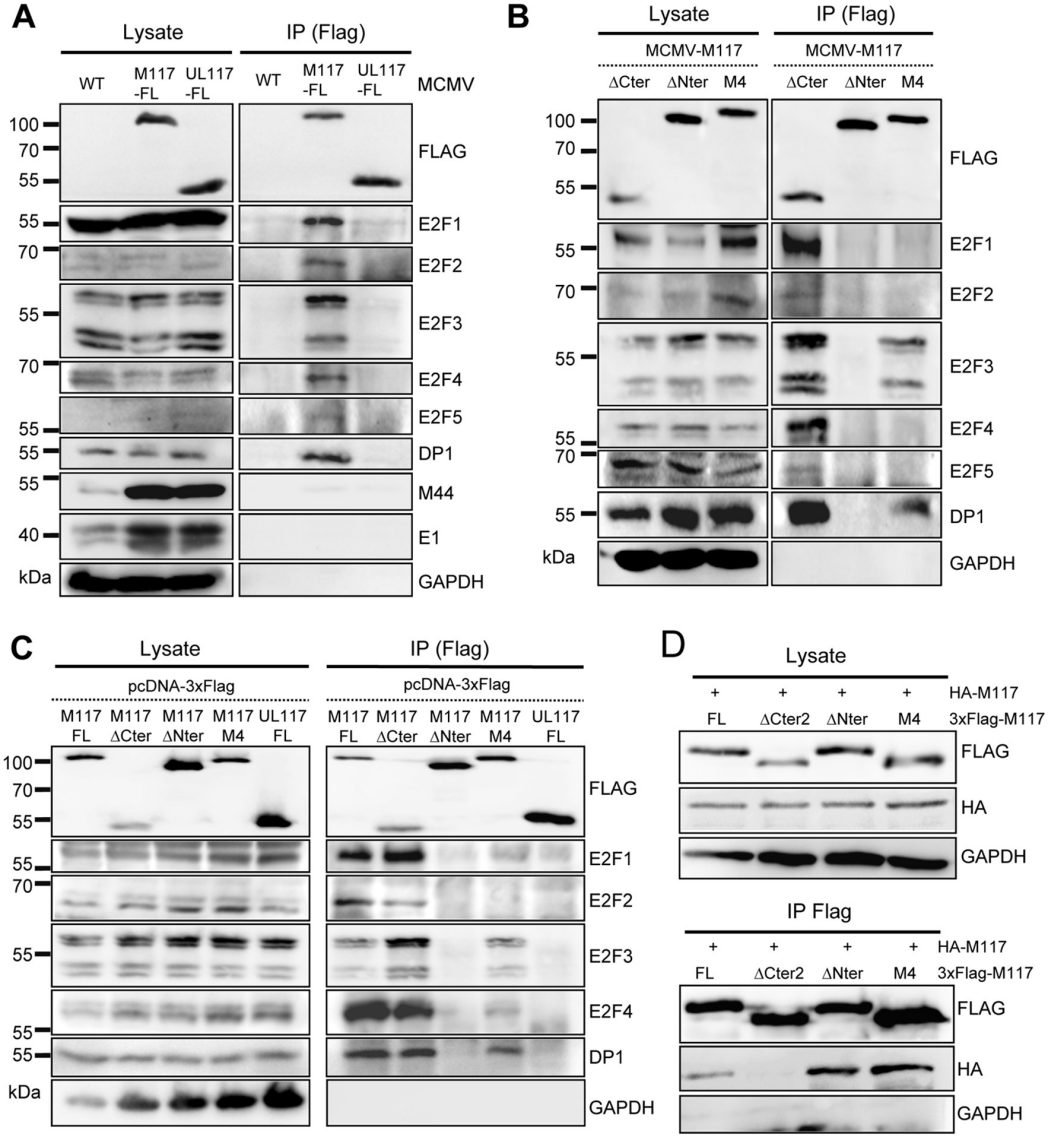
M117 interacts with E2F transcription factors and with itself. (A, B) NIH-3T3 cells were infected with WT MCMV or MCMVs expressing 3xFlag-tagged full-length (FL) M117 or UL117 or mutant M117 proteins (MOI of 3 TCID_50_/cell for 24 hours). Cell lysates were subjected to immunoprecipitation (IP) using an anti-Flag antibody. Co-precipitating proteins were detected by Western blot analysis. (C) NIH-3T3 cells were transfected with pcDNA3 expression plasmids encoding the same 3xFlag-tagged proteins as above. (D) NIH-3T3 cells were co-transfected with pcDNA3 plasmids expressing HA-tagged M117 and 3xFlag-tagged FL or mutant M117. Anti-Flag IP and Western blot analysis were done as above.

To verify the candidates, M117 was immunoprecipitated from MCMV-infected cells using a different antibody (anti-Flag instead of anti-HA) and co-precipitating proteins were analyzed by immunoblot. WT MCMV, which does not express any Flag-tagged protein, and a recombinant virus expressing 3xFlag-tagged UL117 instead of M117 (MCMV-UL117-FL) were used as controls. Immunoprecipitates were separated by SDS-PAGE, blotted, and probed with antibodies specific for all canonical E2F transcription factors (E2F1-5) and DP1. Blots were also probed with antibodies specific for M44 (the viral DNA polymerase processivity factor) and E1 (a major constituent of viral replication compartments). As shown in Fig 5A, neither M44 nor E1 co-precipitated with M117. Comparable results were obtained in repeated experiments. Thus, we could not confirm the M117-M44 interaction and considered M44 as an unspecific interactor in the AP-MS screen. In contrast, all tested members of the E2F transcription factor family interacted with M117: E2F1, E2F3, E2F4, and DP-1 were highly enriched in the immunoprecipitates while E2F2 and E2F5 were detected at lower levels (Fig 5A and S2A Fig). Interestingly, HCMV pUL117 did not interact with any of the E2F factors tested, suggesting that M117 and pUL117 employ different mechanisms to regulate the cell cycle.

To determine which part of M117 is important for the interaction with E2F proteins, N- and C-terminal truncation mutants (ΔNter and ΔCter) were used for co-immunoprecipitation experiments. While deletion of the C-terminal 281 amino acids (aa) did not impair the co-precipitation of E2F transcription factors, deletion of the N-terminal 50 aa was sufficient to abrogate these interactions in MCMV infected cells (Fig 5B). The same interactions were detected when mutant M117 proteins were expressed by plasmid transfection (Fig 5C and S2B Fig), indicating that the presence of other viral proteins is not required for these interactions. Additional 50 aa deletions within the N-terminal half of M117 were constructed (S2C Fig) and tested for their ability to interact with E2F3 and E2F4. The results showed that while aa 51–100 (M117-ΔNter2) were also important for M117 interaction with E2F family members, the following aa 101–200 (M117-ΔNter3 and M117-ΔNter4) did not play any role (S2B Fig). Occasionally, a protein co-precipitating with M117-ΔNter2 was detected with the anti-E2F3 antibody (S2B Fig), but this protein was not seen in other experiments. As the band representing the protein was weak and migrated slightly different than the usual E2F3 isoforms, it is unclear if this band represents an E2F3 isoform or a different cross-reacting protein.

As such large deletions could affect the protein’s conformation, we tested whether an alanine substitution mutation of a highly conserved motif (IPP→AAA, S1 and S2C Figs) would influence the interaction with E2F transcription factors. This mutation, which was named M4, resulted in a loss of interaction with all canonical E2F members except E2F3 and DP1 in MCMV-infected cells (Fig 5B). In plasmid-transfected cells the M117 M4 mutant could still interact very weakly with E2F1 and E2F4 (Fig 5C), suggesting that the M4 mutation strongly impairs the interaction with E2F1 and E2F4, but does not abolish it completely. The M4 mutant retained the ability to bind DP1, albeit to a lesser extent than full-length M117, whereas the M117-ΔNter did not interact with this factor (Fig 5B and 5C). These data suggest that the interaction of M117 with DP1 occurs indirectly via the binding of M117 to E2F-DP heterodimers. Another possibility is that M117 interacts independently with DP1 and E2F3, and that the loss of interaction with DP1 leads indirectly to a loss of interaction with E2F1 and E2F4. Finally, we observed that the C-terminal part of the protein is important for its self-interaction, as a mutant lacking the 117 final amino acids (M117-ΔCter2) could not interact with the full length M117 (Fig 5D).

Taken together, these data show that M117 interacts with E2F transcription factors via its N-terminus, whereas the C-terminus is essential for its self-interaction (dimerization). Moreover, a specific mutation leads to the loss of interaction with specific E2Fs, suggesting a change in the affinity of M117 for specific E2Fs.

### M117 activates the E2F dependent transcription

**Fig 6:**
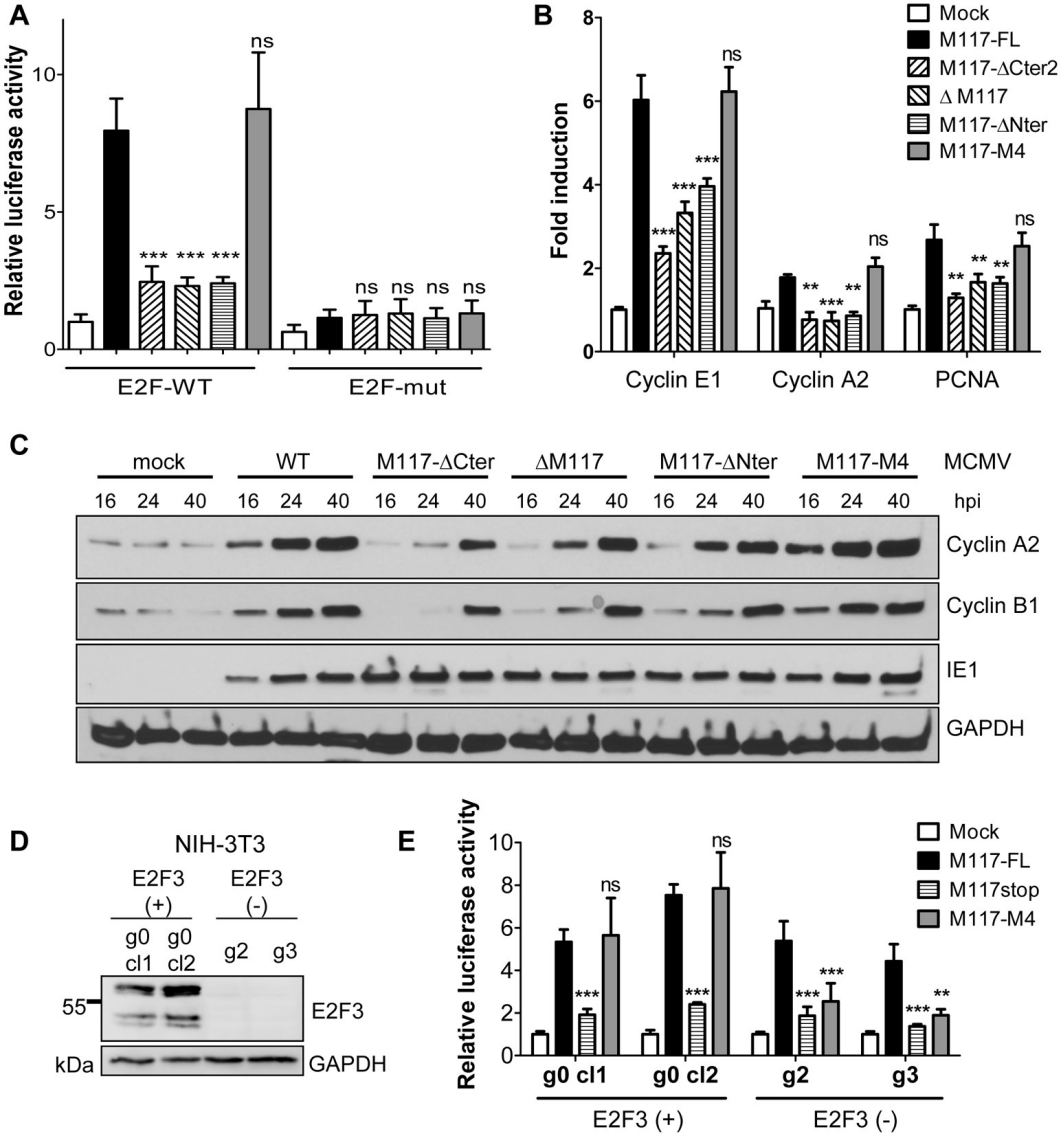
M117 activates E2F-dependent transcription. (A) NIH-3T3 cells were first co-transfected with the reporter plasmid pGL2-Firefly-E2F or pGL2-Firefly-E2Fmut and the control plasmid pRL-Renilla. After 24 hours, cells were infected at an MOI of 3 TCID_50_/cell with viruses expressing full-length (FL) or mutant M117. Cell lysates were harvested 24 hpi and luciferase activities were measured. Mean ±SEM of 3 independent experiments are shown. Firefly luciferase activity was normalized to Renilla luciferase activity and values relative to mock are shown. M117 mutant viruses were compared to M117-FL. (B) NIH-3T3 cells were synchronized by serum starvation for 24 hours, infected at an MOI of 3 TCID_50_/cell (without serum) and harvested at 24 hpi for RNA extraction and qRT-PCR with primers specific for the indicated genes. Mean ±SEM of 4 independent experiments are shown. Transcripts were quantified using the ΔΔCt method and normalized to a housekeeping gene (Gapdh). Values relative to mock-infected cells are shown. M117 mutant viruses were compared to M117-FL. (C) Cells were infected as in (B) and protein lysates were harvested at the indicated times and subjected to Western Blot analysis. (D) Cell lysates of two E2F3-positive (g0 cl1 and cl2) and two E2F3-negative NIH-3T3 cells (g2 and g3) were harvested and subjected to Western Blot analysis. (E) E2F3-positive and negative cells were transfected, infected, and analyzed as in (A). Means ±SEM of 4 independent experiments are shown. ns, not significant; *, p<0.05; **, p<0.01; ***, p<0.001.

The E2F transcription factors have been classified as functional activators or repressors of gene expression [28,56]. As M117 interacted with all canonical members of the E2F family, we wanted to determine the effect of M117 on E2F-dependent transcription. For this purpose, we used a reporter plasmid containing the firefly luciferase gene under the control of an E2F-dependent promoter. NIH-3T3 cells were first co-transfected with this reporter plasmid and a control plasmid expressing Renilla luciferase and then infected either with MCMV-M117-FL or the mutant viruses. Cell lysates were harvested 24 hpi and luciferase activity was measured with a dual luciferase assay. Cells infected with MCMV expressing a 3xFlag-tagged full-length M117 (MCMV-M117-FL) showed an increased firefly luciferase expression compared to uninfected cells, indicating that MCMV induces E2F-dependent transcription (Fig 6A). E2F-dependent luciferase expression was strongly reduced in cells infected by MCMV mutants expressing truncated M117 (M117-ΔNter and M117-ΔCter2) or lacking M117 proteins (M117stop) (Fig 6A), suggesting that both the N-terminus (required for E2F interaction) and the C-terminus (required for self-interaction) of M117 are needed to activate E2F-dependent transcription. Surprisingly, the MCMV-M117-M4 mutant virus activated E2F-dependent transcription to a similar extent as did MCMV-M117-FL, suggesting that the E2F family member E2F3 is a very potent transcription factor under these conditions. Mutation of the E2F binding site of the reporter plasmid abrogated the induction of luciferase (Fig 6A), confirming that M117 induced luciferase expression in an E2F-dependent manner.

To verify the results obtained with the luciferase reporter assay, we measured the impact of MCMV infection on the transcription of selected cellular genes known to be regulated by E2Fs [57]. NIH-3T3 cells were synchronized in G1 and infected with WT and mutant viruses. Total RNA was extracted at 24 hpi and used to quantify Cyclin E1, Cyclin A2, and PCNA transcripts by quantitative RT-PCR (qRT-PCR). Similarly to what we had observed with the E2F reporter assay (Fig 6A), infection with an MCMV expressing a full-length M117 (MCMV-M117-FL) or the M4 mutant induced the expression of the E2F target genes. This induction was significantly reduced when cells were infected with the M117-ΔNter, M117-ΔCter2, or the M117stop mutant (Fig 6B).

We also analyzed the protein levels of Cyclin A2 and Cyclin B1, representative E2F target gene products, by immunoblot. Unfortunately, none of the antibodies against mouse Cyclin E1 that we tested worked reliably in our hands. Hence we were unable to determine Cyclin E1 protein levels. As shown in Fig 6C, Cyclin A2 and B1 protein levels were upregulated upon infection with MCMV-M117-FL or MCMV-M117-M4. In contrast, Cyclin A2 and B1 induction was much weaker after infection with the M117-ΔCter2, M117-ΔNter or the ΔM117 mutants. The protein expression data are consistent with the qRT-PCR results.

To examine the importance of E2F3 in the M117-mediated E2F dependent transcription, we generated E2F3 knockout (KO) NIH-3T3 cells using CRISPR/Cas9 genome editing. Two cell clones lacking E2F3 expression and two control clones (Fig 6D) were used in an E2F-dependent luciferase reporter assay. As shown in Fig 6E, luciferase induction by MCMV-M117-FL was similar in E2F3-positive and negative cells, indicating that E2F3 is not required for MCMV to induce E2F-dependent transcription. In contrast, luciferase induction by MCMV-M117-M4 was strongly impaired only in E2F3-negative cells, but reached comparable levels to the MCMV-M117-FL in E2F3-positive cells, indicating that the interaction of M117 with E2F3 is sufficient to induce E2F-dependent transcription.

Taken together, the data in Fig 6 show that M117 is necessary for the activation of E2F-regulated genes in MCMV-infected cells. However, M117 might not be sufficient as M117 expression by plasmid transfection did not reliably activate an E2F-dependent luciferase reporter, and the intranuclear distribution of M117 protein was dependent on the presence of other viral factors (Fig 3). Thus, any functional analyses of M117 done solely in transfection experiments should be interpreted with caution. We concluded that additional factors present in MCMV-infected cells might be required for M117-dependent activation of E2F target genes.

### M117 is required for viral dissemination in vivo

**Fig 7:**
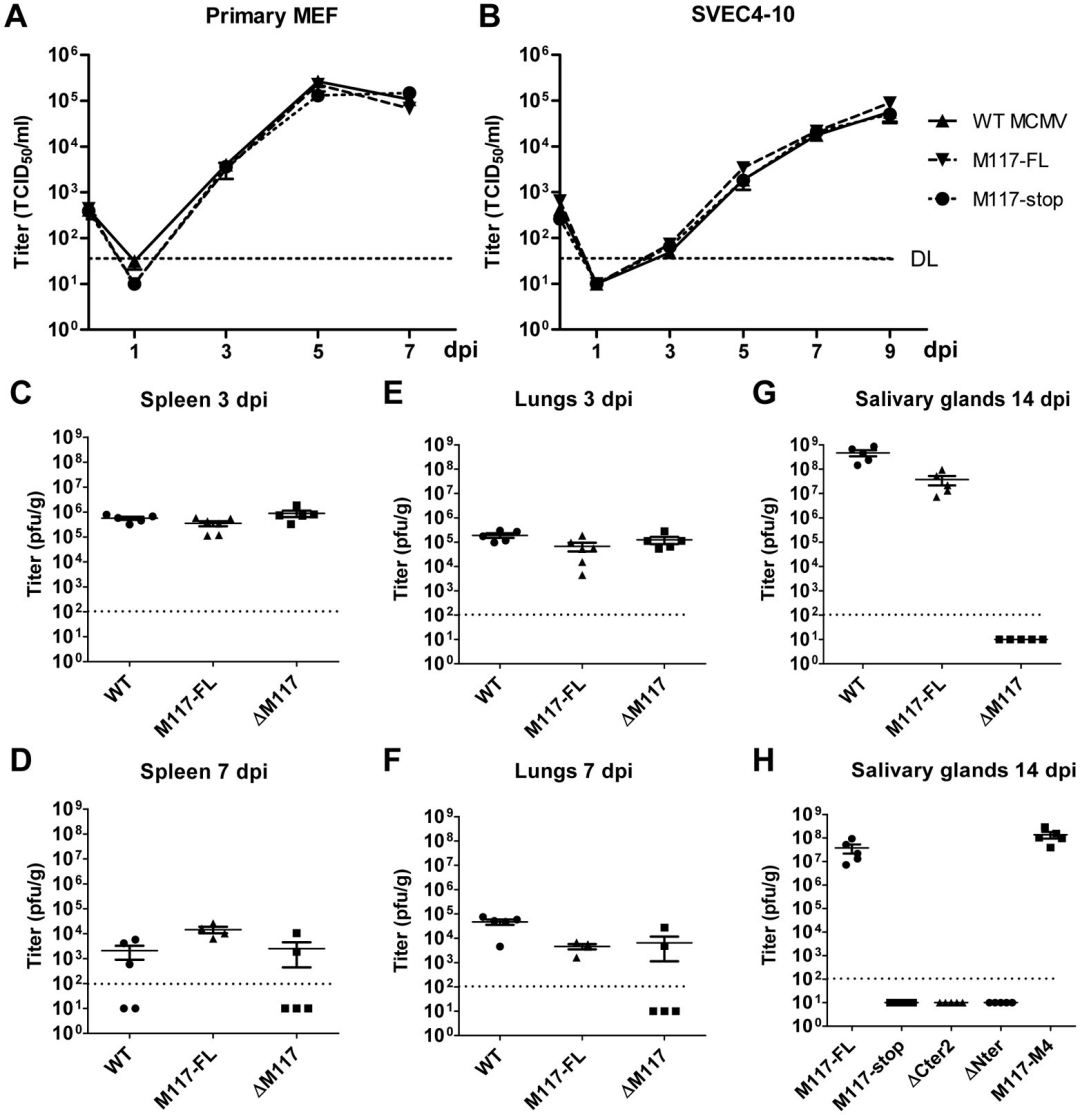
M117 is required for MCMV dissemination to the salivary glands. Primary MEF (A) or SVEC4-10 endothelial cells (B) were infected with WT and mutant MCMV at an MOI 0.02 TCID_50_/cell. Supernatants of infected cells were harvested at the indicated time point after infection and titrated. The experiments were done in triplicate. Mean ±SEM are shown (C-H) Mice were infected intraperitoneally with 10^5^ PFU. After 3, 7, and 14 days viral titers in the spleen (C, D), lungs (E, F), or salivary glands (G, H) were determined. Mean titers ±SEM are shown. DL: detection limit.

Considering the clear effects of M117 on E2F-dependent gene expression (Fig 6) and cell cycle regulation in immortalized fibroblasts (Fig 4), we were surprised to see that MCMV-M117stop replicated with the same kinetics as WT MCMV in different cells such as primary MEFs (non-immortalized cells), and SVEC4-10 endothelial cells (Fig 7A and 7B). Other M117 mutant viruses also showed no signs of impaired replication in these murine cells (S3 Fig). However, it remained possible that M117 mutant viruses had a more subtle or cell type-dependent replication and dissemination disadvantage that becomes apparent only during infection in vivo. To test this, BALB/c mice were infected intraperitoneally with WT MCMV, MCMV-ΔM117, and MCMV-3xFlag-M117 (MCMV-M117-FL), which served as a revertant of the MCMV-ΔM117 mutant. Viral titers in the spleen and lungs were determined on day 3 and 7, and salivary gland titers were determined on day 14 post infection. Again, we did not observe any obvious replication defect for MCMV-ΔM117 in the acute phase of MCMV infection (Fig 7C–7F). However, on day 14 post infection salivary gland titers of the MCMV-ΔM117 mutant were massively reduced to levels below the detection limit (Fig 7G).

To exclude that the salivary gland phenotype of the MCMV mutant lacking M117 was caused by an unspecific effect of the deletion on neighboring genes, we infected mice with MCMV-M117stop. We also included MCMV N- and C-terminal M117 truncation mutants as well as the MCMV-M117-M4 mutant. On day 14 post infection, salivary gland titers of MCMV-M117stop, ΔNter, and ΔCter2 were below the detection limit (Fig 7H), thus confirming that a loss of M117 function was responsible for the in vivo attenuation. Strikingly, the MCMV-M117-M4 mutant attained salivary gland titers comparable to those of the MCMV-M117-FL (Fig 7H), suggesting that the interaction with E2F3 (the only E2F interaction retained by M117-M4 as shown in Fig 5B) is sufficient not only for the activation of E2F target genes (Fig 6) but also for dissemination to or replication in the salivary glands (Fig 7H).

### Reduction of E2F activity facilitates MCMV replication in human RPE-1 cells

**Fig 8:**
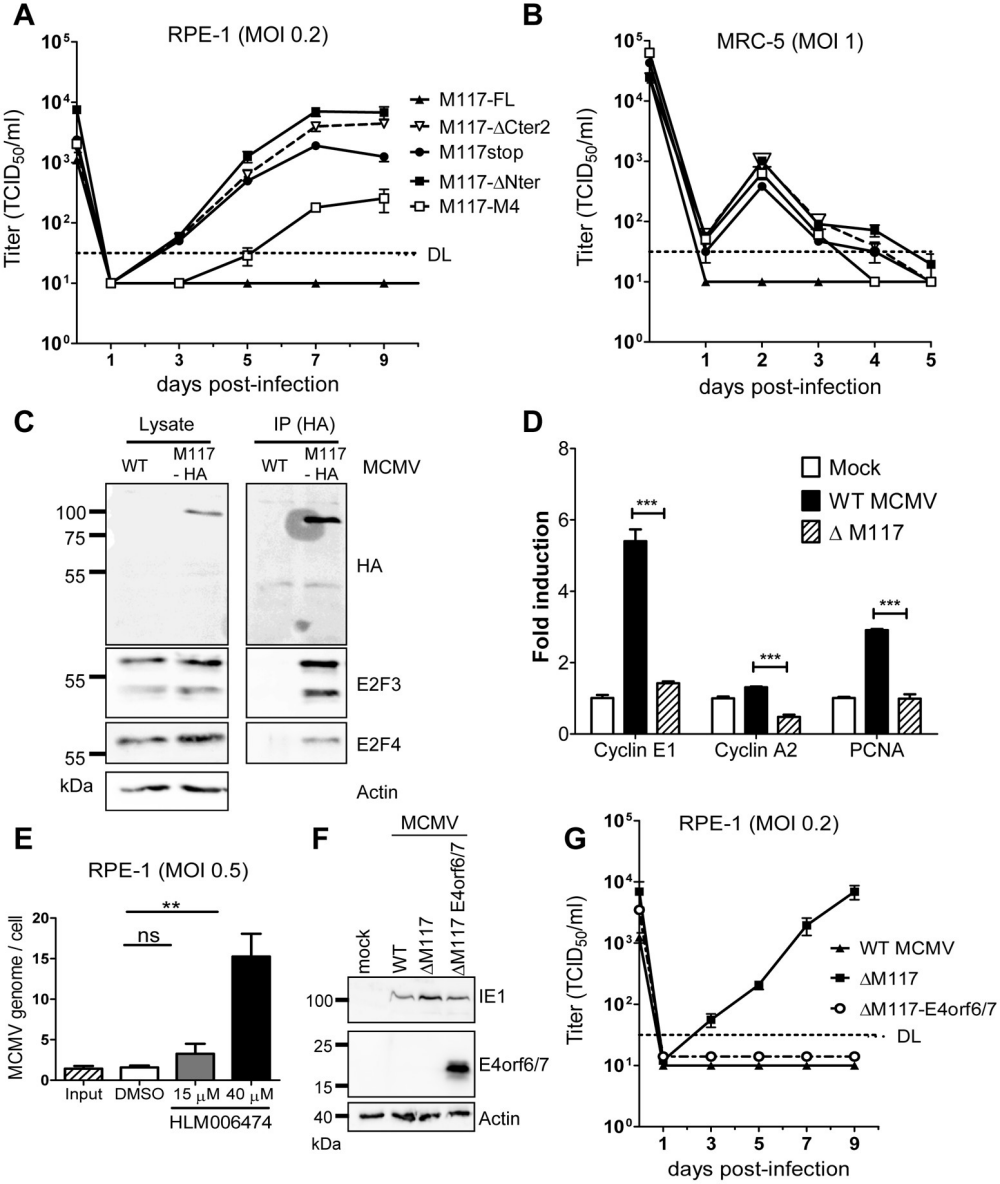
E2F activation is detrimental for MCMV replication in human cells. (A) Multistep growth kinetic in human RPE-1 cells. Cells were infected with MCMV-M117-FL and the recombinant viruses at an MOI of 0.2 TCID_50_/cell. Supernatant of infected cells were harvested at the indicated time point and titrated. The experiments were done in triplicate. Means ±SEM are shown. (B) Multistep growth kinetic in human embryonic lung fibroblasts (MRC-5). Cells were infected with MCMV-M117-FL and the recombinant viruses at an MOI of 1 TCID_50_/cell. Supernatants of infected cells were harvested at the indicated time points and titrated with centrifugal enhancement. The experiments were done in triplicate. Means ±SEM are shown. (C) RPE-1 cells were infected with WT MCMV or MCMV-M117-HA. Cell lysates were subjected to immunoprecipitation (IP) using an anti-HA antibody. Co-precipitating proteins were detected by Western blot analysis. (D) RPE-1 cells were synchronized by serum starvation for 48 hours, infected at an MOI of 3 TCID_50_/cell (without serum) and harvested at 24 hpi for RNA extraction and qRT-PCR with primers specific for the indicated genes. Means ±SEM of 3 independent experiments are shown. Transcripts were quantified using the ΔΔCt method and normalized to a housekeeping gene (Gapdh). (E) RPE-1 cells were infected at an MOI of 0.5 TCID_50_/cell with MCMV-GFP. Three hpi, the medium was removed and the E2F inhibitor HLM006474 was added. At 3 hpi (Input) or after 3 days, the cell lysates were harvested for DNA extraction and viral DNA quantification by qPCR. MCMV genome copies were normalized to β-actin copies. Means ±SEM are shown. (F) RPE-1 cells were infected at an MOI of 2 TCID_50_/cell for 24h. Cell lysates were harvested 24 hpi and subjected to SDS-PAGE. (G) Multistep growth kinetic in human RPE-1 cells. Cells were infected with WT MCMV, MCMV-ΔM117 and MCMV-ΔM117-E4orf6/7 at an MOI of 0.2 TCID_50_/cell. Supernatants of infected cells were harvested at the indicated time points and titrated. The experiments were done in triplicate. Means ±SEM are shown. DL, detection limit; ns, not significant; *, p<0.05; **, p< 0.01; ***, p<0.001.

In the beginning of this study, we characterized M117 as a host range determinant: mutations causing the expression of C-terminally truncated M117 proteins allowed MCMV replication in human RPE-1 cells (Fig 1). However, it has remained unknown whether expression of the N-terminal part of M117 was required for MCMV replication in human RPE-1 cells or whether any loss-of-function mutation would have the same effect. To resolve this question we infected RPE-1 cells with a set of M117 mutants and determined viral replication kinetics. As expected, MCMV-M117-FL did not replicate in RPE-1 cells. By contrast, MCMV-M117stop and both the N- and C-terminal M117 truncation mutants grew to high titers and with very similar kinetics (Fig 8A). The MCMV-M117-M4 mutant showed an intermediate phenotype (i.e., it replicated slower and reached lower titers), possibly because it retained the ability to interact with E2F3 (Fig 5B and 5D) and activated E2F-responsive promoters (Fig 6). As RPE-1 cells are telomerase-immortalized cells, we tested whether the virus mutants were also able to replicate in primary human cells. We have previously shown that human cell-adapted MCMVs can also replicate in human embryonic lung fibroblasts (MRC-5 cells), although to substantially lower titers than in RPE-1 cells [13,14]. Therefore, we tested whether mutation of M117 is sufficient to allow MCMV replication in MRC-5 cells. As expected, MCMV-M117-FL did not replicate in human fibroblasts, whereas all M117 mutants replicated to low titers in MRC-5 (Fig 8B), indicating that the presence of a fully active M117 is detrimental for MCMV replication in human cells.

To understand whether the function of M117 was similar in mouse and human cells, we performed experiments similar to the ones described above. First, we checked if M117 was able to interact with the human E2F members. M117-HA was immunoprecipitated from RPE-1 infected cells and co-precipitating proteins were analyzed by immunoblot. WT MCMV, which does not express any HA-tagged proteins, was used as a control. Immunoprecipitates were separated by SDS-PAGE, blotted, and probed with antibodies specific for two E2F transcription factors, E2F3 and E2F4. As shown in Fig 8C, E2F3 and E2F4 co-precipitated with HA-M117, suggesting a similar interaction pattern of M117 in human and mouse cells. Next, we looked at the activation of the E2F-dependent gene expression by qRT-PCR. RPE-1 cells were synchronized in G1 and infected with WT MCMV and MCMV-ΔM117 viruses. Total RNA was extracted at 24 hpi and used to quantify Cyclin E1, Cyclin A2, and PCNA transcripts. Similarly to what was observed in mouse cells, infection with a WT MCMV induced the expression of the E2F target genes Cyclin E1 and PCNA, and to a lower extent of Cyclin A2. However, this induction was significantly reduced in absence of M117 for all the genes tested (Fig 8D).

Finally, we wanted to clarify whether the M117-mediated E2F activation or another hitherto unknown function of M117 precludes MCMV replication in human cells. To address this question, we tested whether HLM006474, a pan-E2F inhibitor [58], could facilitate MCMV replication in human RPE-1 cells. This inhibitor was shown to block the E2F DNA-binding activity [58], but did not affect the interaction of M117 with E2Fs (S4 Fig). Unfortunately, prolonged treatment with HLM006474 was not possible due to cell toxicity [58]. To restrict the duration of inhibitor treatment, RPE-1 cells were infected with WT MCMV-GFP, and treated with HLM006474 or solvent (DMSO) for 3 days. Viral DNA replication was then measured by qPCR. As shown in Fig 8E, MCMV DNA replication was increased substantially in the presence of the pan-E2F inhibitor HLM006474.

As E4orf6/7 from AdV 5 shares functional similarities with M117, such as the interaction with E2Fs and activation of the E2F-dependent transcription [41,59], we constructed a recombinant virus by inserting the E4orf6/7 gene into MCMV-ΔM117. Expression of E4orf6/7 by MCMV (Fig 8F) abrogated the ability of MCMV-ΔM117 to replicate in human RPE-1 cells (Fig 8G). These results support the concept that E2F activation is detrimental for MCMV replication in human cells and explain why loss-of-function mutations in M117 facilitate MCMV replication in these cells.

## Discussion

In this work we characterize the properties, functions and cellular interacting partners of M117, a previously uncharacterized MCMV protein. M117 has sequence similarity to pUL117, its presumed homolog in HCMV, and the two proteins show functional similarities: Both are expressed with early kinetics, localize to viral replication compartments, and contribute to the blockage of host DNA synthesis. However, there are important differences: Inactivation of UL117 in HCMV lead to a profound replication defect of the virus in cell culture [55], whereas the absence of M117 did not affect MCMV replication in vitro (Fig 7). Moreover, the two proteins have a different mechanism of action. While pUL117 inhibits cellular DNA synthesis by inhibiting chromatin loading of the minichromosome maintenance complex [26], MCMV M117 interacts with E2F transcription factors and activates E2F-dependent gene transcription. This activation could be the result of a recruitment of the M117-E2F complex to specific promoters. Alternatively, the binding of M117 to E2Fs could alter the interaction of E2Fs with repressors such as pRB, ARF or EAPP. This mechanism is not shared with HCMV pUL117 (Fig 5) but appears to be similar to the one described for AdV 5 E4orf6/7. The E4orf6/7 protein binds to all classical E2Fs [39] and activates E2F-dependent transcription of the viral E2 genes as well as cellular genes [40,41] by inducing a dimerization of E2F factors [40,60]. M117 shares several of these features such as the ability to interact with several E2F family members and the self-interaction that is needed to activate target gene transcription. Moreover, a recombinant MCMV expressing E4orf6/7 instead of M117 had the same replication phenotype in human cells as WT MCMV (Fig 8G), indicating that E4orf6/7 can substitute for M117 under these conditions. Interestingly, the M117 M4 mutation abrogated the interaction of M117 with most E2F proteins (Fig 5). The 3 aa mutated in the M117 M4 mutant appear to be part of a larger motif that is conserved in the rat CMV homologs (R117 and E117), but only to a very limited extent in HCMV UL117 (S1 Fig). This could explain the inability of UL117 to interact with E2F proteins (Fig 5A). It is also possible that E2F binding requires a three-dimensional structure (consisting of two or more protein domains) that is not conserved in UL117.

Similar to E4orf6/7, the absence of M117 does not affect viral replication in cell culture, suggesting that another viral protein can compensate for the loss of M117. In the case of E4orf6/7, the AdV E1A protein provides the redundant function by inducing pRb phosphorylation [61,62]. We propose that a similar mechanism could operate during MCMV infection. A strong candidate as provider of the redundant function is the MCMV protein kinase, M97, as its HCMV homolog, pUL97, can bind and phosphorylate pRb, thereby relieving its inhibitory effect on the E2Fs [63,64]. This hypothesis remains to be confirmed. However, our observation that M117 mutant viruses (MCMV-M117stop and MCMV-ΔNter) are still able to moderately induce E2F-dependent gene expression in infected cells (Fig 6A and 6B) supports the hypothesis that other viral factors are involved in the activation of E2Fs.

Induction of E2F-dependent transcription by M117 (Fig 6) is expected to accelerate cell cycle progression and push the cell cycle toward the S phase. However, we also found that M117 helps to block cell cycle progression, as seen when cells infected with an M117-deficient MCMV were able to progress toward the G2/M phase (Fig 4). These apparently contradictory findings can be reconciled taking into account that CMV replication induces a DNA damage response, which can lead to a cell cycle arrest at the G1/S transition [65,66]. As many E2F target genes belong to the DNA repair machinery, their sustained E2F-dependent transcription is beneficial for those cells experiencing replication stress [67,68]. We hypothesize that MCMV uses M117 to push the host cell towards S phase, which provides a favorable environment for viral DNA replication. The resulting constant E2F activation, together with the viral DNA replication, increases replication stress, which leads to cell cycle arrest. However, cells infected with an MCMV lacking a functional M117 can still enter S phase with the help of compensatory proteins (for example, those that phosphorylate pRb,) and activate E2F-dependent transcription to a lesser extent. However, inhibitory mechanisms exist to turn off E2F-dependent transcription [69], thus reducing the replication stress experienced by infected cells and preventing cell cycle arrest.

When we analyzed the ability of M117 mutant MCMVs to replicate and disseminate in vivo, we observed a profound drop in the salivary gland titers at 14 dpi (Fig 7), indicating that viral dissemination to or replication within the salivary glands is defective in the absence of a functional M117. At 14 dpi, the virus is largely cleared from other organs (spleen, lungs) while the salivary glands remain important sites for viral persistence. The reason why M117 is important at this stage of the infection remains unclear. As putative E2F binding sites have been detected in the promoters of cytokines and chemokines [70–72], one could speculate that the E2F transcription factors regulate the expression of cytokines or chemokines necessary for viral dissemination to or replication in the salivary glands. Interestingly, the MCMV-M117-M4 mutant activated E2F-dependent transcription (Fig 6) and disseminated to the salivary glands in vivo (Fig 7) to a comparable extent as WT MCMV did. These findings were somewhat surprising as the M4 mutant only retained the ability to interact efficiently with E2F3, but not with the other canonical E2F family members. Thus, the activation of E2F3 appears to be sufficient for viral replication and dissemination in vivo. This conclusion is consistent with the results of genetic studies in mice: knockout mice lacking several E2F members were viable as long as at least one E2F3 isoform was present [73,74].

Finally, we demonstrated that M117 acts as a host range determinant. Activation of the E2F-dependent transcription by M117 or by E4orf6/7 is detrimental for the replication of MCMV in human RPE-1 cells, and inhibiting this activation, either by deleting M117 or by adding an inhibitor, allows the virus to replicate in human cells (Figs 1 and 8). The reason why E2F activation is detrimental to MCMV replication in human but not murine cells remains to be investigated. There is probably no simple answer to this question as E2F transcription factors affect the expression of many different genes. Some of them could have antiviral activities against MCMV that cannot be counteracted by the virus. E2F functions detrimental for viral replication might include the upregulation of Cyclin A2, which has been shown to inhibit the immediate early gene expression of HCMV [23]. However, in our experiments, we observed only a low induction of Cyclin A2 in WT MCMV infected cells (Fig 8C), an observation that argues against a crucial role of Cyclin A2. Nonetheless, we cannot completely reject this possibility as the time point selected in human infected cells (24 hpi) could be too early to observe a strong induction of Cyclin A2 gene expression. The detrimental effect of M117 in human cells could also be caused by differences in the target genes induced by the E2F factors in human vs. murine cells.

Altering the viral host range by adapting a virus to cells of a different host can raise biosafety concerns if a virus with zoonotic potential is involved. This is not the case for the cytomegaloviruses: They are highly adapted to their specific host and cause persistent infections only because of numerous immune evasion mechanisms [75], many of which do not function properly in a foreign host. Hence the zoonotic potential of the CMVs is very low, and the CMVs are not among the pathogens that could be involved in “dual use research of concern” [76].

In conclusion, this study identified MCMV M117 as a new viral E2F regulator, which is important for viral fitness in vivo. M117 also acts as a host range factor that prevents MCMV replication in human RPE-1 and MRC-5 cells. Further work needs to be done to understand the function of this protein in cross-species infection as well as the differences in E2F-mediated gene regulation between human and mouse cells.

## Materials and methods

### Ethics statement

All animal experiments were performed according to the recommendations and guidelines of the FELASA (Federation for Laboratory Animal Science Associations) and Society of Laboratory Animals (GV-SOLAS) and approved by the institutional review board and local authorities (Behörde für Gesundheit und Verbraucherschutz, Amt für Verbraucherschutz, Freie und Hansestadt Hamburg, reference number 123/16).

### In vivo experiments

Six to 8 week-old BALB/c female mice (Janvier laboratories) were infected intraperitoneally with 10^5^ PFU MCMV per mouse as described [77]. Organs were harvested on day 3 and 7 (spleen and lungs) and day 14 (salivary glands) post infection, homogenized, and used to determine organ titers by plaque assay on M2-10B4 cells.

### Cell culture and viruses

All cells were maintained in complete Dulbecco’s modified Eagle medium (DMEM) supplemented with 10% fetal calf serum (FCS) and 100 IU Penicillin/100 μg Streptomycin at 37°C and 5% CO_2_. RPE-1 cells are human telomerase reverse transcriptase immortalized human retinal pigment epithelial cells (ATCC CRL-4000). MRC-5 cells are primary human embryonic lung fibroblasts (ATCC CCL-171). Murine NIH-3T3 fibroblasts (CRL-1658), SVEC4-10 endothelial cells (CRL-1658), and M2-10B4 bone marrow stromal cells (CRL-1972) were obtained from the ATCC. Murine 10.1 fibroblasts are spontaneously immortalized MEFs from BALB/c mice [78] obtained from Thomas Shenk (Princeton University, USA). Primary MEFs were isolated from 13.5 day old C56BL/6 embryos following standard procedures [77]. The GFP-expressing (MCMV-GFP) and the “repaired” WT MCMV Smith strain (pSM3fr-MCK-2fl) strain have been described previously [79,80]. All viruses were propagated in 10.1 fibroblasts. For high MOI infections, a centrifugal enhancement (1000 × *g*, 30 min) was used. For replication kinetics, cells were infected in six-well dishes. After 4 h, cells were washed twice with phosphate-buffered saline (PBS), and fresh medium was added. Supernatants were harvested at different times post infection for virus titration on 10.1 cells using the median tissue culture infective dose (TCID_50_) method.

### BAC mutagenesis and sequencing

Mutations within the M117 locus were introduced by *en passant* mutagenesis [81] into the MCMV-GFP or the WT MCMV (pSM3fr-MCK-2fl) bacterial artificial chromosome (BAC), which was used as parental wildtype (WT MCMV) genome. AdV 5 E4orf6/7 was PCR amplified from pcDNA-E4orf6/7 (kindly provided by Thomas Dobner, Heinrich Pette Institute, Hamburg) and introduced into the nonessential m02-m06 region of MCMV (pSM3fr-MCK-2fl) by using the pReplacer system [8]. To reconstitute infectious virus from MCMV BACs, purified BAC DNA was transfected into 10.1 fibroblasts using Polyfect (Qiagen). The integrity and the absence of unintended mutations of the mutant BACs based on the backbone of the WT MCMV were verified by Illumina sequencing of the entire BACs (MCMV-3xFlag-M117-FL, M117stop, ΔM117, ΔCter, ΔCter2, ΔNter). All other mutant BACs based on the backbone of MCMV-GFP (MCMV-M117mut-h1 and -h3, HA-M117, M117-HA and m117.1-HA) were analyzed by sequencing of the region of interest and analysis of restriction fragment patterns.

### Plasmids

The M117 coding sequence was PCR-amplified from the MCMV Smith genome using a forward primer containing a HindIII restriction site and the 3xFlag sequence and a reverse primer containing an MfeI restriction site. The PCR product was cleaved with HindIII and MfeI and inserted into expression plasmid pcDNA3 (Invitrogen) digested with HindIII and EcoRI. Deletions or substitutions of nucleotides were performed by PCR-driven overlap extension [82]. Transfection of NIH-3T3 cells with expression plasmids was done using Polyfect (Qiagen) according to the manufacturer’s protocol.

### Generation of E2F3 knockout NIH-3T3 cells

The lentiviral CRISPR/Cas9 vector pSicoR-CRISPR-PuroR was used to generate E2F3 KO clones essentially as described [83]. Briefly, the E-CRISPR design tool (www.e-crisp.org) was used to design three guideRNAs that were cloned individually in the lentiviral vector: g1, GATGGTCTAAAGACCCCCAA; g2, GGATCTGAACAAGGCAGCAG; and g3, GGACCTCAAACTGTTAACCG. Lentiviruses were generated using standard third-generation packaging vectors in HEK-293T cells. Then, NIH-3T3 cells were transduced with an E2F3 lentiviral CRISPR/Cas9 or an empty vector (g0) in the presence of polybrene (Sigma). The cells were selected with 2.5 μg/mL puromycin (Sigma). Polyclonal cultures were subcultured to obtain single cell clones for each gRNA, and E2F3 protein expression was evaluated for each clone by Western blot analysis.

### Quantitative real-time PCR (qPCR)

To quantify transcripts, synchronized NIH-3T3 or RPE-1 cells were infected at a MOI of 3 TCID_50_/cell. Total RNA was extracted from infected cells using the innuPREP RNA Mini Kit (Analytik Jena) and contaminating DNA was removed using the TURBO DNA-free Kit (Ambion). cDNA was synthesized from 1μg of the extracted RNA by using the RevertAid H Minus Reverse Transcriptase, oligo-dT primers, and the RNase inhibitor RiboLock (Thermo Fisher Scientific). qPCR was performed on an ABI PRISM 7900HT Fast Real-Time PCR System (Applied Biosystem) using 10 ng of cDNA, the SybrGreen real time PCR Mastermix (Life technologies). To amplify mouse transcripts, the following primers were used: Gapdh (CCCACTCTTCCACCTTCGATG and GTCCACCACCCTGTTGCTGTAG), PCNA (CACGTATATGCCGAGACCTTAGC and CTCCACTTGCAGAAAACTTCACC), Cyclin A2 (GAGGGCCATCCTTGTGGACT and CACAGCCAAATGCAGGGTCT), and Cyclin E1 (CAGAGCAGCGAGCAGGAGA and GCAGCTGCTTCCACACCACT). For the amplification of human transcripts, the following primers were used: hPCNA (GGCACTCAAGGACCTCATCAAC and GTGAGCTGCACCAAAGAGACG), hGAPDH (CCCACTCCTCCACCTTTGACG and GTCCACCACCCTGTTGCTGTAG), hCyclin A2 (TGCTGGAGCTGCCTTTCATT and TGAAGGTCCATGAGACAAGGCT), and hCyclin E1 (TACACCAGCCACCTCCAGACAC and CCTCCACAGCTTCAAGCTTTTG). Transcripts were quantified using the ΔΔCt method and normalized to a housekeeping gene (Gapdh).

For MCMV genome quantification, total DNA was extracted from MCMV-infected RPE-1 cells using an InnuPREP DNA Mini Kit (Analytik Jena). One hundred ng of DNA was subjected to qPCR in order to quantify MCMV genome copies (primers ACTAGATGAGCGTGCCGCAT and TCCCCAGGCAATGAACAATC) and human β-actin gene copies (primers GCTGAGGCCCAGTTCTAAAT and TTCAAGTCCCATCCCAGAAAG).

### Cell cycle analysis

NIH-3T3 cells were synchronized for 48 h by serum starvation (DMEM/ 0.5% FCS) and then infected with MCMV in the presence of 10% FCS. After 1 h, cells were washed and new media containing 50 μM Ganciclovir was added. To measure both DNA content and viral protein expression, cells were first permeabilized by incubation in 75% ethanol for at least 12 h at 4°C. Afterwards, cells were stained with antibodies against IE1 (CROMA 101, from Stipan Jonjic, University of Rijeka, Croatia) and mouse-IgG-Alexa647, before staining for DNA content with propidium iodide and analyzed by flow cytometry as previously described [22].

### E2F reporter assay

The luciferase reporter plasmids were kindly provided by Matthias Truss (Charité Universitätsmedizin Berlin, Germany). They are based on a promoter/enhancer-less pGL2-basic (Promega) modified by insertion of a TATA box and an E2F binding site [84]. Plasmids pGL2-E2F-WT and pGL2-E2F-mut carry a single wildtype (CGCGCC) or mutant (AAAGCC) E2F binding site, respectively. NIH-3T3 cells were seeded in 12-wells plates (1×10^5^ cells/well) and transfected with 1 μg pGL2-E2F and 100 ng of pRL-Renilla (Promega) using Polyfect (Qiagen). After 6 h the medium was exchanged, and after 24 h cells were infected at a MOI of 3 TCID_50_/cell. At 24 hpi, cells were harvested and luciferase activity was measured on a FLUOstar Omega reader (BMG Labtech) using a Dual Luciferase Reporter Assay (Promega) and according to the manufacturer’s protocol.

### Immunocytochemistry

NIH-3T3 cells were seeded onto glass coverslips coated for 30 min with 0.4% gelatin/PBS. On the following day, cells were infected with MCMV-3xFlagM117 at an MOI of 1 TCID_50_/cell or transfected with pcDNA3 expression plasmids using Polyfect (Qiagen). Cells were fixed in methanol for 10 min at -20°C, washed with PBS, and blocked with PBS containing 1% gelatin. Incubations with the antibodies were carried out for 1 h at room temperature (RT) in 1% gelatin/PBS. Nuclei were stained for 10 min at RT with DRAQ5 (BioStatus) diluted 1:1000 in PBS. Coverslips were mounted on microscope slides with Aqua-Poly/Mount (Polysciences) and subjected to confocal laser scanning microscopy (cLSM).

### Antibodies

The following antibodies were used: monoclonal antibodies against glyceraldehyde-3-phosphate dehydrogenase (GAPDH) (14C10; Cell Signaling), β-actin (AC-74; Sigma), HA (3F10; Roche), FLAG (M2, Sigma-Aldrich), DP1 (TFD-10; Santa Cruz), and Cyclin B1 (GNS-1; Santa Cruz). Antibodies against MCMV gB (M55.01), IE1 (CROMA101), and M112-113 (CROMA103) were provided by Stipan Jonjic (University of Rijeka, Rijeka, Croatia), anti-M44 (3B9.22A) was provided by Lambert Loh, University of Saskatchewan, Canada. The monoclonal antibody RSA3 against AdV 5 E4orf6/7 [85] was kindly provided by Thomas Dobner (Heinrich Pette Institute, Hamburg). Polyclonal rabbit antibodies against E2F1 (C-20), E2F2 (C-20), E2F3 (C-18), E2F4 (C-20), E2F5 (E-19), and Cyclin A2 (C-19) were purchased from Santa Cruz. Secondary antibodies coupled to horseradish peroxidase (HRP) were purchased from DakoCytomation or Jackson ImmunoResearch. Secondary antibodies coupled to Alexa-488 or Alexa-555 were from ThermoFischer.

### Immunoprecipitation and immunoblot analysis

Cells grown in 6-well plates were infected at an MOI of 3 TCID_50_/cell. At 24 hpi, cells were lysed in a NP-40 buffer (50 mM Tris, 150 mM NaCl, 1% Nonidet P-40, and Complete Mini protease inhibitor cocktail [Roche]). Insoluble material was removed by centrifugation. After preclearing with protein G Sepharose (PGS, GE Healthcare), Flag-tagged M117 protein was precipitated with anti-Flag and PGS. Precipitates were washed 3 times with buffer 1 (1 mM Tris pH 7.6, 150 mM NaCl, 2 mM EDTA, 0.2% NP-40), 2 times with buffer 2 (1 mM Tris pH 7.6, 500 mM NaCl, 2mM EDTA, 0.2% NP-40) and 1 time with buffer 3 (10mM Tris pH 7.6), eluted by boiling in SDS-PAGE sample buffer (125 mM Tris pH 6.8, 4% SDS, 20% glycerol, 10% β-mercaptoethanol, 0.002% bromophenol blue) and subjected to SDS-PAGE and immunoblotting.

For immunoblot analysis, cells were lysed in NP-40 buffer or in SDS-PAGE sample buffer. Equal amounts of protein (NP-40 lysis) or equal volumes (sample buffer) were subjected to SDS-PAGE followed by transfer to a nitrocellulose membrane (Amersham). Proteins of interest were detected with protein-specific primary antibodies and HRP-coupled secondary antibodies by enhanced chemiluminescence (Amersham) supplemented with 10% of Lumigen TMA-6 (Bioquote Limited). All immunoblots showed are representatives of 3 or more experiments, except for S2A and S4 Figs, which are representatives of 2 experiments.

### SILAC and AP-MS

For stable isotope incorporation, cells were grown in DMEM (high-glucose) SILAC medium supplemented with 10% dialyzed FCS, 4 mM glutamine, 100 IU penicillin/100 μg streptomycin in the presence of either ^13^C_6_,^15^N_2_-lysine / ^13^C_6_,^15^N_4_-arginine (SILAC heavy) or unlabeled lysine / arginine (SILAC light). All SILAC media were filtered before usage and stored at 4°C for up to 6 month. To ensure complete incorporation of SILAC amino acids (≥ 97%) cells were grown for at least 5 passages in SILAC medium. For immunoprecipitation, two populations were generated, one grown in light and the other in heavy media. Either the light or the heavy labelled cells were infected with the HA-M117, whereas the other labelled population was infected with the WT MCMV. After lysis in NP-40 buffer, HA tagged-M117 proteins were precipitated with rat anti-HA Affinity Matrix antibody (clone 3F10, Roche). Precipitates were washed 3 times with minimal washing buffer (50 mM Tris, 150 mM NaCl, 10% (v/v) glycerol, pH 7.5) and eluted by boiling in elution buffer (1% (w/v) SDS in 50 mM Tris, 150 mM NaCl, pH 7.5). Afterwards, the samples were mixed in a light-to-heavy ratio of 1:1 for subsequent sample processing.

### LC-MS sample preparation and analysis

Cysteines were reduced in the presence of 10 mM DTT at 56 °C for 30 min and alkylated using 20 mM IAA for 30 min at RT in the dark. Proteins were precipitated by diluting the samples 1:10 with ice-cold ethanol (-40 °C) and incubation at -40 °C for 1 h. Precipitated proteins were spun down at 20,000 x g at 4 °C for 40 min and the pellet was washed with 50 μl ice-cold acetone (-40 °C). After 15 minutes of incubation at -40 °C proteins were spun down for 15 min at 20,000 x g and 4 °C. The sediment was solubilized in 7.5 μl 2 M guanidinium hydrochloride (GuHCl) and 10-fold diluted using 50 mM ABC and 1 mM CaCl_2_. Proteins were digested by adding 5 μl of a 0.1 μg/μl trypsin solution (Sigma-Aldrich T-1426) followed by incubation at 37°C for 12.5 h. The reaction was stopped by adding TFA to a final concentration of 1%. Samples were dried in a vacuum centrifuge, reconstituted in 0.1% TFA and subjected to LC-MS.

NanoLC-MS/MS was conducted using a U3000 HPLC system online coupled to a Q Exactive HF (both Thermo Scientific). Samples were loaded in 0.1% TFA on a C18 trap column (HiChrom ACE, 100 μm x 2 cm, 5 μm particles) and separated on a C18 main column (HiChrom ACE, 75 μm x 30 cm, 5 μm particles) using a 60 min linear gradient-program from 2.5% to 35% ACN in the presence of 0.1% FA at 60°C and a flow rate of 270 nL/min. The column effluent was introduced to the MS by nanoESI using a PicoTip emitter (new objectives) operated at 1.5 kV. The MS was operated in positive ion-mode using a top15 HCD data-dependant acquisition method with a resolution of 60,000 for MS and a resolution of 15,000 for MS/MS. The normalized collision energy was set to 27 and only ions with an assigned charge state of 2–4 were selected for fragmentation. Automatic gain control target values were set to 10^6^ and 5x10^4^ with maximum ion injection times of 120 and 250 ms for MS and MS/MS, respectively. The dynamic exclusion was set to 12 sec and the m/z = 371.10124 lock mass was used for internal calibration.

### Data analysis

A database search was done using Mascot v2.4.1 implemented in Proteome Discoverer v1.4 against a merged database comprising all Uniprot entries of *Mus musculus* and all Uniprot/TreEMBL entries of MCMV strain Smith and K181 (January 2013, 16,799 target sequences). The decoy search option was enabled and mass error tolerances were set to 10 ppm and 0.02 Da for MS and MS/MS. A maximum of 2 missed cleavages was allowed, Oxididation of Met and heavy labelled Arg/Lys was set as variable modification and carbamidomethylation of Cys was set as static modifications. Results were filtered for ≤ 1% FDR at the PSM level and feature quantification was done within a 2 ppm mass precision window using the precursor ion quantifier. PSM tables were exported and further processed with Microsoft Excel and R to calculate the number of unique peptides per protein in each condition and calculate median-normalized heavy/light ratios. Proteins identified with ≥ 2 unique peptides and a log2 ratio ≥ 3 (8-fold enrichment) in both replicates were considered as potential M117 interaction partners.

### Statistical analysis

All the statistical analyses were performed with the GraphPad Prism 5.0 Software. A one-way ANOVA with Tukey’s multiple comparison post test was used for the analysis of the qPCR on genomic DNA. A two-way ANOVA with Bonferroni post test was used for the analysis of the luciferase reporter assays and qPCR on transcripts.

## Supporting information

S1 Fig Alignment of M117 homologs in different virus species. The amino acid sequences of the M117 and its homologs in the English and Maastricht isolates of RCMV (E117 and R117, respectively), HCMV (UL117), and Chimpanzee CMV (UL117-C) were aligned using Multalin software [86]. Identical and similar amino acid residues are shaded black and grey, respectively. A predicted bipartite NLS in M117 and the M4 motif are indicated. GenBank accession numbers: AQQ81376 (M117), AFX83425 (E117), AAF99208 (R117), P16770 (UL117), AAM00747 (UL117-C).
(TIF)

S2 Fig Requirement of the M117 N-terminus for E2F interaction. (A) NIH-3T3 cells were infected with MCMVs expressing 3xFlag-tagged M117 or UL117 full-length (FL) or mutant proteins. Cell lysates were subjected to immunoprecipitation (IP) using an anti-E2F3 antibody. Co-precipitating M117 proteins were detected by Western blot analysis. (B) NIH-3T3 cells were transfected with pcDNA3 expression plasmids encoding 3xFlag-tagged M117 proteins with N-terminal 50 aa deletions. Cell lysates were subjected to immunoprecipitation (IP) using an anti-Flag antibody. Co-precipitating E2F proteins were detected by Western blot analysis. (C) Schematic of the M117 mutants used in this study. ΔCter, deletion of aa 285–565; ΔCter2: frameshift of aa 449–481; ΔNter, deletion of aa 1–50 aa; ΔNter2, deletion of aa 51–100; ΔNter3, deletion of aa 101–150; ΔNter4, deletion of aa 151–200; M4, IPP→AAA substitution at positions 59–61.
(TIF)

S3 Fig Mutations in M117 do not affect viral replication in mouse cells. Primary MEF (A) or SVEC4-10 endothelial cells (B) were infected with WT and mutant MCMV at an MOI 0.02 TCID_50_/cell. Supernatants of infected cells were harvested at the indicated times post infection and titrated. The experiments were done in triplicate. Mean ±SEM are shown. DL, detection limit.
(TIF)

S4 Fig HLM006474 does not inhibit M117–E2F interactions. Human RPE-1 cells were infected with mutant MCMVs at an MOI of 2 TCID_50_/cell. Three hours post infection, cells were treated with HLM006474 (+) for 24 or 48 hours or left untreated (-). Cell lysates were subjected to immunoprecipitation using an anti-Flag antibody. Co-precipitating proteins were detected by Western blot analysis. *, antibody heavy chain.
(TIF)
