# Supplementary figures and images for: Activation of E2F-dependent transcription by the mouse cytomegalovirus M117 protein affects the viral host range
Source: PLoS Pathog. 2018 Dec 10;14(12):e1007481. doi: 10.1371/journal.ppat.1007481 (PMC6301716; doi:10.1371/journal.ppat.1007481)

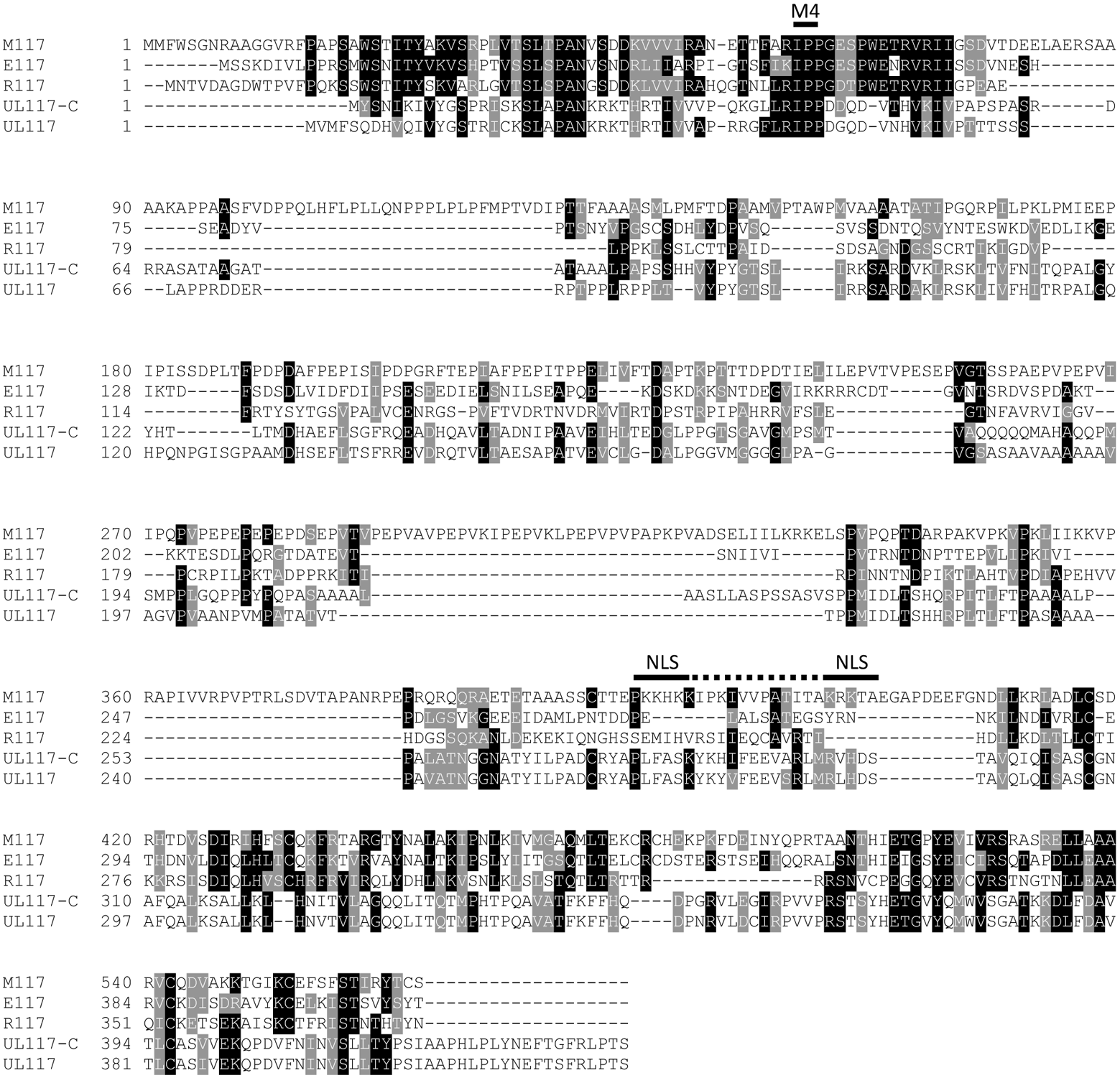

Supplement: S1 Fig — The amino acid sequences of the M117 and its homologs in the English and Maastricht isolates of RCMV (E117 and R117, respectively), HCMV (UL117), and Chimpanzee CMV (UL117-C) were aligned using Multalin software [86]. Identical and similar amino acid residues are shaded black and grey, respectively. A predicted bipartite NLS in M117 and the M4 motif are indicated. GenBank accession numbers: AQQ81376 (M117), AFX83425 (E117), AAF99208 (R117), P16770 (UL117), AAM00747 (UL117-C). (TIF) [file ppat.1007481.s001.tif]

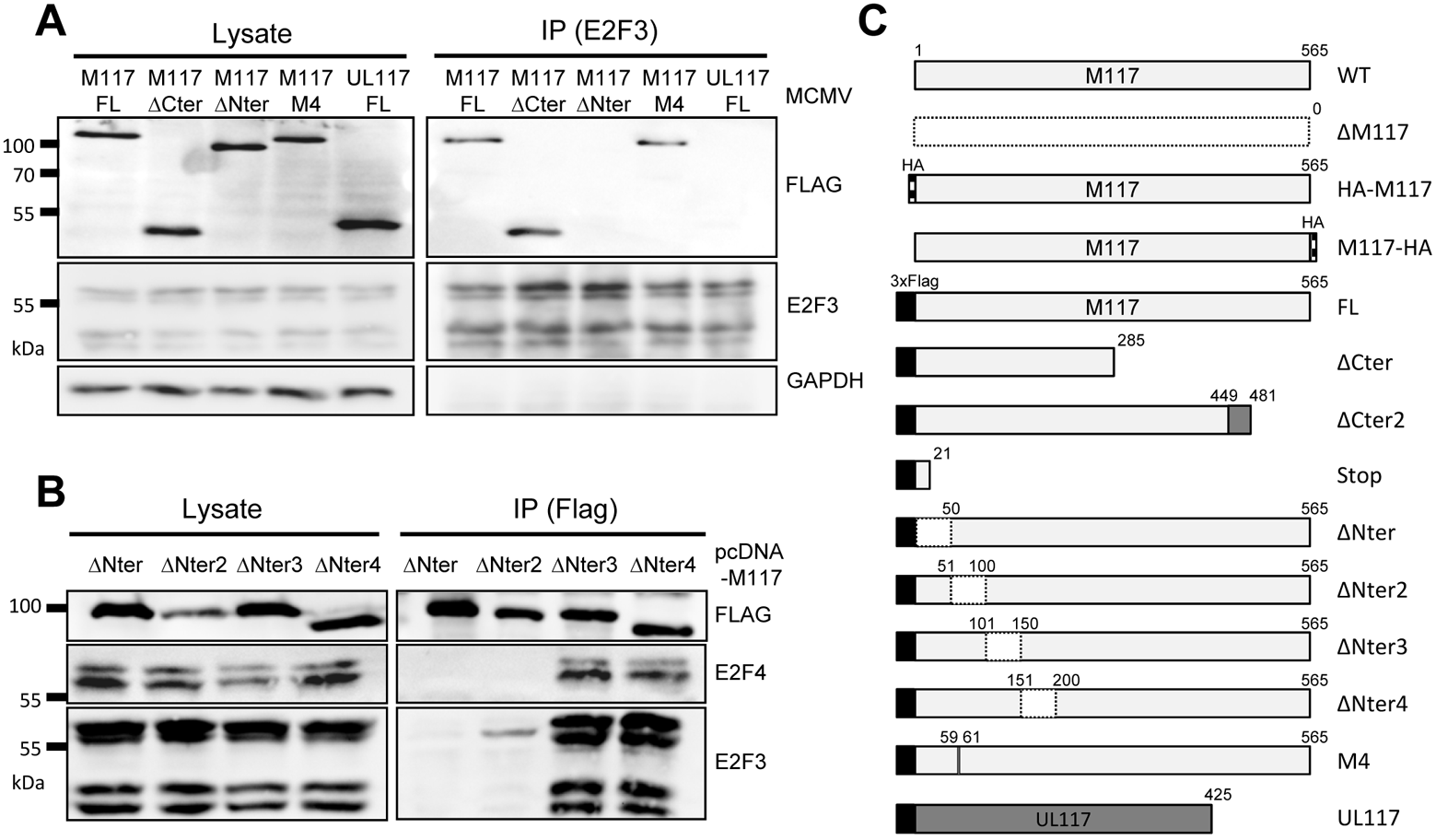

Supplement: S2 Fig — (A) NIH-3T3 cells were infected with MCMVs expressing 3xFlag-tagged M117 or UL117 full-length (FL) or mutant proteins. Cell lysates were subjected to immunoprecipitation (IP) using an anti-E2F3 antibody. Co-precipitating M117 proteins were detected by Western blot analysis. (B) NIH-3T3 cells were transfected with pcDNA3 expression plasmids encoding 3xFlag-tagged M117 proteins with N-terminal 50 aa deletions. Cell lysates were subjected to immunoprecipitation (IP) using an anti-Flag antibody. Co-precipitating E2F proteins were detected by Western blot analysis. (C) Schematic of the M117 mutants used in this study. ΔCter, deletion of aa 285–565; ΔCter2: frameshift of aa 449–481; ΔNter, deletion of aa 1–50 aa; ΔNter2, deletion of aa 51–100; ΔNter3, deletion of aa 101–150; ΔNter4, deletion of aa 151–200; M4, IPP→AAA substitution at positions 59–61. (TIF) [file ppat.1007481.s002.tif]

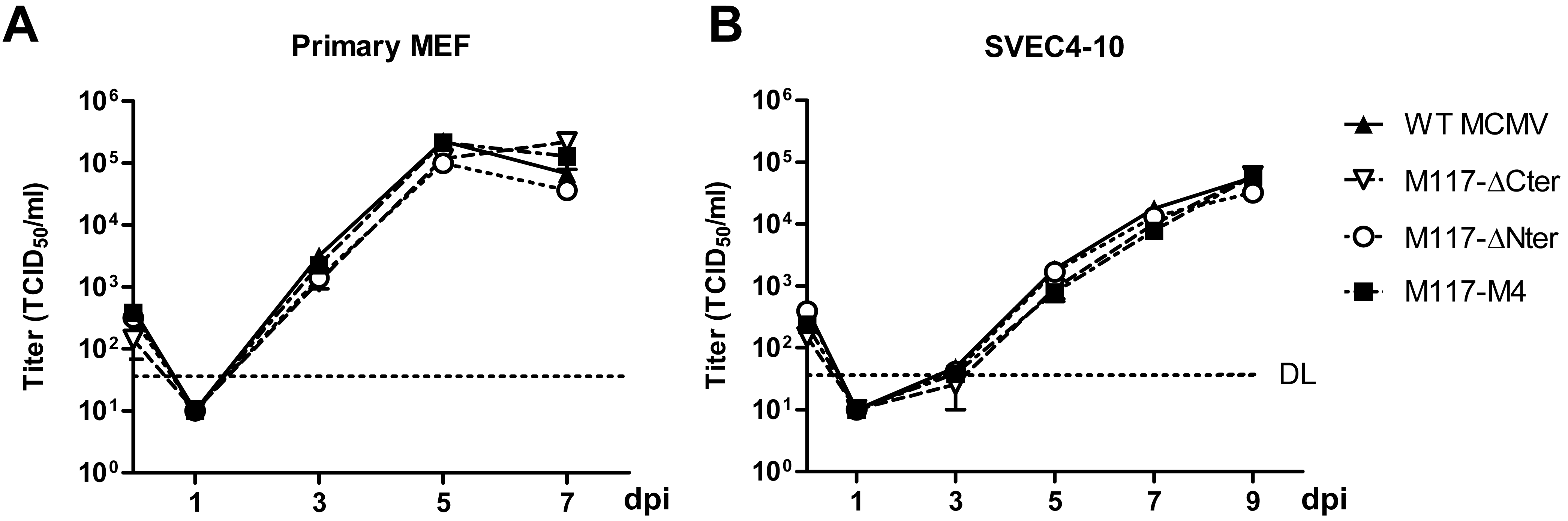

Supplement: S3 Fig — Primary MEF (A) or SVEC4-10 endothelial cells (B) were infected with WT and mutant MCMV at an MOI 0.02 TCID50/cell. Supernatants of infected cells were harvested at the indicated times post infection and titrated. The experiments were done in triplicate. Mean ±SEM are shown. DL, detection limit. (TIF) [file ppat.1007481.s003.tif]

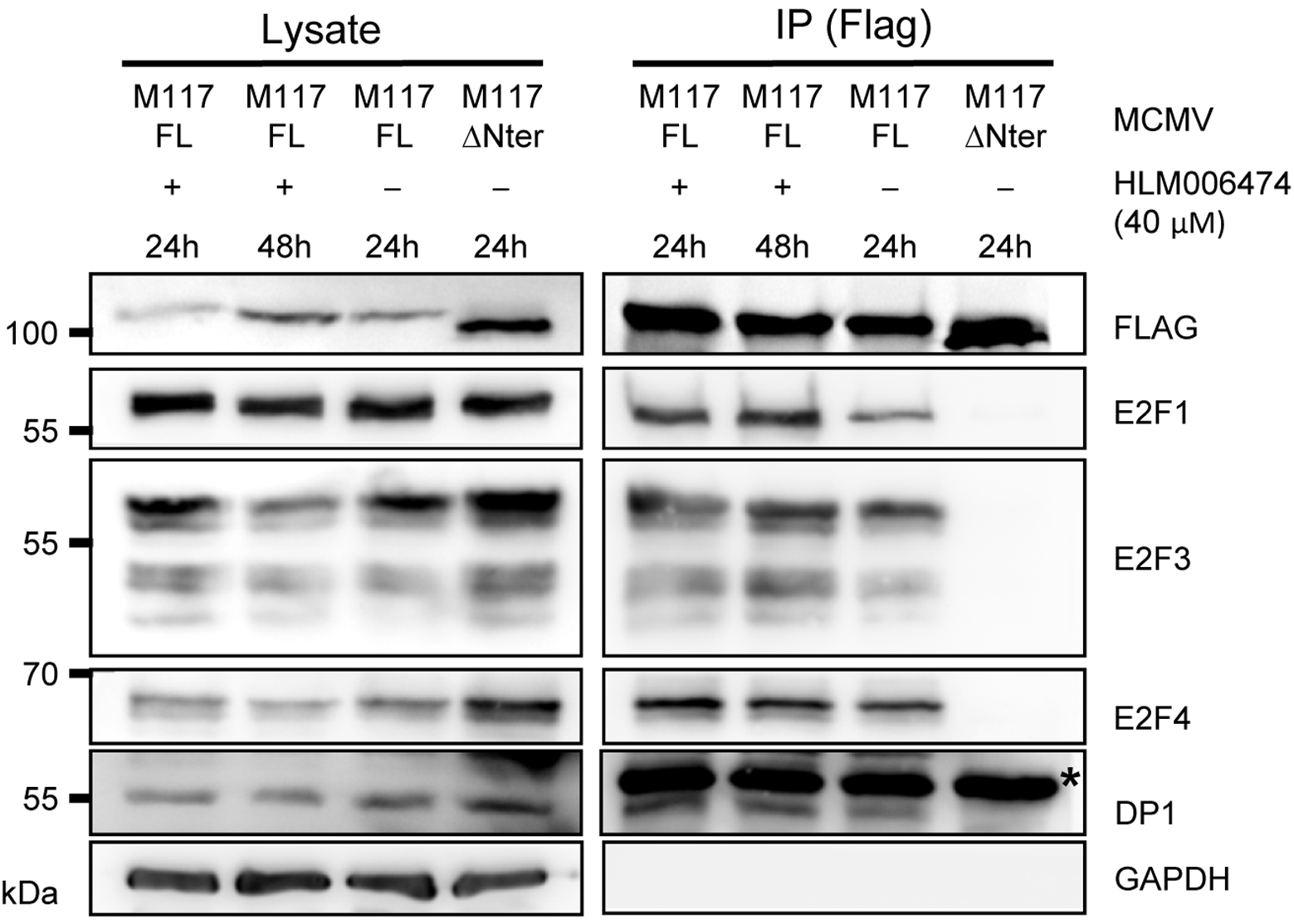

Supplement: S4 Fig — Human RPE-1 cells were infected with mutant MCMVs at an MOI of 2 TCID50/cell. Three hours post infection, cells were treated with HLM006474 (+) for 24 or 48 hours or left untreated (-). Cell lysates were subjected to immunoprecipitation using an anti-Flag antibody. Co-precipitating proteins were detected by Western blot analysis. *, antibody heavy chain. (TIF) [file ppat.1007481.s004.tif]
